# Copy number variants as modifiers of breast cancer risk for *BRCA1*/*BRCA2* pathogenic variant carriers

**DOI:** 10.1038/s42003-022-03978-6

**Published:** 2022-10-06

**Authors:** Christopher Hakkaart, John F. Pearson, Louise Marquart, Joe Dennis, George A. R. Wiggins, Daniel R. Barnes, Bridget A. Robinson, Peter D. Mace, Kristiina Aittomäki, Irene L. Andrulis, Banu K. Arun, Jacopo Azzollini, Judith Balmaña, Rosa B. Barkardottir, Sami Belhadj, Lieke Berger, Marinus J. Blok, Susanne E. Boonen, Julika Borde, Angela R. Bradbury, Joan Brunet, Saundra S. Buys, Maria A. Caligo, Ian Campbell, Wendy K. Chung, Kathleen B. M. Claes, GEMO Study Collaborators, EMBRACE Collaborators, Marie-Agnès Collonge-Rame, Jackie Cook, Casey Cosgrove, Fergus J. Couch, Mary B. Daly, Sita Dandiker, Rosemarie Davidson, Miguel de la Hoya, Robin de Putter, Capucine Delnatte, Mallika Dhawan, Orland Diez, Yuan Chun Ding, Susan M. Domchek, Alan Donaldson, Jacqueline Eason, Douglas F. Easton, Hans Ehrencrona, Christoph Engel, D. Gareth Evans, Ulrike Faust, Lidia Feliubadaló, Florentia Fostira, Eitan Friedman, Megan Frone, Debra Frost, Judy Garber, Simon A. Gayther, Andrea Gehrig, Paul Gesta, Andrew K. Godwin, David E. Goldgar, Mark H. Greene, Eric Hahnen, Christopher R. Hake, Ute Hamann, Thomas V. O. Hansen, Jan Hauke, Julia Hentschel, Natalie Herold, Ellen Honisch, Peter J. Hulick, Evgeny N. Imyanitov, SWE-BRCA Investigators, kConFab Investigators, HEBON Investigators, Klaartje van Engelen, Marijke R. Wevers, Claudine Isaacs, Louise Izatt, Angel Izquierdo, Anna Jakubowska, Paul A. James, Ramunas Janavicius, Esther M. John, Vijai Joseph, Beth Y. Karlan, Zoe Kemp, Judy Kirk, Irene Konstantopoulou, Marco Koudijs, Ava Kwong, Yael Laitman, Fiona Lalloo, Christine Lasset, Charlotte Lautrup, Conxi Lazaro, Clémentine Legrand, Goska Leslie, Fabienne Lesueur, Phuong L. Mai, Siranoush Manoukian, Véronique Mari, John W. M. Martens, Lesley McGuffog, Noura Mebirouk, Alfons Meindl, Austin Miller, Marco Montagna, Lidia Moserle, Emmanuelle Mouret-Fourme, Hannah Musgrave, Sophie Nambot, Katherine L. Nathanson, Susan L. Neuhausen, Heli Nevanlinna, Joanne Ngeow Yuen Yie, Tu Nguyen-Dumont, Liene Nikitina-Zake, Kenneth Offit, Edith Olah, Olufunmilayo I. Olopade, Ana Osorio, Claus-Eric Ott, Sue K. Park, Michael T. Parsons, Inge Sokilde Pedersen, Ana Peixoto, Pedro Perez-Segura, Paolo Peterlongo, Timea Pocza, Paolo Radice, Juliane Ramser, Johanna Rantala, Gustavo C. Rodriguez, Karina Rønlund, Efraim H. Rosenberg, Maria Rossing, Rita K. Schmutzler, Payal D. Shah, Saba Sharif, Priyanka Sharma, Lucy E. Side, Jacques Simard, Christian F. Singer, Katie Snape, Doris Steinemann, Dominique Stoppa-Lyonnet, Christian Sutter, Yen Yen Tan, Manuel R. Teixeira, Soo Hwang Teo, Mads Thomassen, Darcy L. Thull, Marc Tischkowitz, Amanda E. Toland, Alison H. Trainer, Vishakha Tripathi, Nadine Tung, Klaartje van Engelen, Elizabeth J. van Rensburg, Ana Vega, Alessandra Viel, Lisa Walker, Jeffrey N. Weitzel, Marike R. Wevers, Georgia Chenevix-Trench, Amanda B. Spurdle, Antonis C. Antoniou, Logan C. Walker

**Affiliations:** 1 Department of Pathology and Biomedical Science, University of Otago, Christchurch, New Zealand; 2 QIMR Berghofer Medical Research Institute, Brisbane, Queensland Australia; 3 School of Public Health, University of Queensland, Brisbane, Australia; 4 Centre for Cancer Genetic Epidemiology, Department of Public Health and Primary Care, University of Cambridge, Cambridge, UK; 5 Department of Medicine, University of Otago, Christchurch, New Zealand; 6 Canterbury Regional Cancer and Haematology Service, Canterbury District Health Board, Christchurch Hospital, Christchurch, New Zealand; 7 Department of Biochemistry, School of Biomedical Sciences, University of Otago, Dunedin, New Zealand; 8 Department of Medical and Clinical Genetics, University of Helsinki, Helsinki, Finland; 9 Fred A. Litwin Center for Cancer Genetics, Lunenfeld-Tanenbaum Research Institute of Mount Sinai Hospital, Toronto, ON Canada; 10 Department of Molecular Genetics, University of Toronto, Toronto, ON Canada; 11 Department of Breast Medical Oncology, University of Texas MD Anderson Cancer Center, Houston, TX USA; 12 Unit of Medical Genetics, Department of Medical Oncology and Hematology, Fondazione IRCCS Istituto Nazionale dei Tumori (INT), Milan, Italy; 13 Hereditary cancer Genetics Group, Vall d’Hebron Institute of Oncology, Vall d’Hebron Hospital Campus, Barcelona, Spain; 14 Department of Medical Oncology, Vall d’Hebron Hospital Universitari, Vall d’Hebron Barcelona Hospital Campus, Barcelona, Spain; 15 Department of Pathology, Landspitali University Hospital, Reykjavik, Iceland; 16 BMC (Biomedical Centre), Faculty of Medicine, University of Iceland, Reykjavik, Iceland; 17 Clinical Genetics Research Lab, Department of Cancer Biology and Genetics, Memorial Sloan Kettering Cancer Center, New York, NY USA; 18 Department of Clinical Genetics, University of Groningen, University Medical Center Groningen, Groningen, The Netherlands; 19 Department of Clinical Genetics, Maastricht University Medical Center, Maastricht, The Netherlands; 20 Department of Clinical Genetics, Odense University Hospital, Odence C, Denmark; 21 Center for Integrated Oncology (CIO), Faculty of Medicine and University Hospital Cologne, University of Cologne, Cologne, Germany; 22 Center for Molecular Medicine Cologne (CMMC), Faculty of Medicine and University Hospital Cologne, University of Cologne, Cologne, Germany; 23 Center for Familial Breast and Ovarian Cancer, Faculty of Medicine and University Hospital Cologne, University of Cologne, Cologne, Germany; 24 Department of Medicine, Abramson Cancer Center, Perelman School of Medicine at the University of Pennsylvania, Philadelphia, PA USA; 25 Hereditary Cancer Program, Catalan Institute of Oncology (ICO), ONCOBELL-IDIBELL-IGTP, CIBERONC, Barcelona, Spain; 26 Department of Medicine, Huntsman Cancer Institute, Salt Lake City, UT USA; 27 SOD Genetica Molecolare, University Hospital, Pisa, Italy; 28 Peter MacCallum Cancer Center, Melbourne, Victoria Australia; 29 Sir Peter MacCallum Department of Oncology, The University of Melbourne, Melbourne, Victoria Australia; 30 Departments of Pediatrics and Medicine, Columbia University, New York, NY USA; 31 Centre for Medical Genetics, Ghent University Hospital, Gent, Belgium; 32 Service de Génétique Biologique, CHRU de Besançon, Besançon, France; 33 Sheffield Clinical Genetics Service, Sheffield Children’s Hospital, Sheffield, UK; 34 Gynecologic Oncology, Translational Therapeutics, Department of Obstetrics and Gynecology, Ohio State University Comprehensive Cancer Center, Columbus, OH USA; 35 Department of Laboratory Medicine and Pathology, Mayo Clinic, Rochester, MN USA; 36 Department of Clinical Genetics, Fox Chase Cancer Center, Philadelphia, PA USA; 37 Department of Clinical Genetics, Queen Elizabeth University Hospital, Glasgow, UK; 38 Molecular Oncology Laboratory, CIBERONC, Hospital Clinico San Carlos, IdISSC (Instituto de Investigación Sanitaria del Hospital Clínico San Carlos), Madrid, Spain; 39 Oncogénétique, Institut de Cancérologie de l’Ouest siteRené Gauducheau, Saint Herblain, France; 40 Cancer Genetics and Prevention Program, University of California San Francisco, San Francisco, CA USA; 41 Area of Clinical and Molecular Genetics, Vall d’Hebron Hospital Universitari, Vall d’Hebron Barcelona Hospital Campus, Barcelona, Spain; 42 Department of Population Sciences, Beckman Research Institute of City of Hope, Duarte, CA USA; 43 Basser Center for BRCA, Abramson Cancer Center, University of Pennsylvania, Philadelphia, PA USA; 44 Clinical Genetics Department, St Michael’s Hospital, Bristol, UK; 45 Nottingham Clinical Genetics Service, Nottingham University Hospitals NHS Trust, Nottingham, UK; 46 Centre for Cancer Genetic Epidemiology, Department of Oncology, University of Cambridge, Cambridge, UK; 47 Department of Clinical Genetics and Pathology, Laboratory Medicine, Skåne University Hospital, Lund, Sweden; 48 Division of Clinical Genetics, Department of Laboratory Medicine, Lund University, Lund, Sweden; 49 Institute for Medical Informatics, Statistics and Epidemiology, University of Leipzig, Leipzig, Germany; 50 LIFE - Leipzig Research Centre for Civilization Diseases, University of Leipzig, Leipzig, Germany; 51 Division of Evolution and Genomic Sciences, School of Biological Sciences, Faculty of Biology, Medicine and Health, University of Manchester, Manchester Academic Health Science Centre, Manchester, UK; 52 North West Genomics Laboratory Hub, Manchester Centre for Genomic Medicine, St Mary’s Hospital, Manchester University NHS Foundation Trust, Manchester Academic Health Science Centre, Manchester, UK; 53 Institute of Medical Genetics and Applied Genomics, University of Tübingen, Tübingen, Germany; 54 Molecular Diagnostics Laboratory, INRASTES, National Centre for Scientific Research ‘Demokritos’, Athens, Greece; 55 The Susanne Levy Gertner Oncogenetics Unit, Chaim Sheba Medical Center, Ramat Gan, Israel; 56 Sackler Faculty of Medicine, Tel Aviv University, Ramat Aviv, Israel; 57 Clinical Genetics Branch, Division of Cancer Epidemiology and Genetics, National Cancer Institute, Bethesda, MD USA; 58 Cancer Risk and Prevention Clinic, Dana-Farber Cancer Institute, Boston, MA USA; 59 Center for Bioinformatics and Functional Genomics and the Cedars Sinai Genomics Core, Cedars-Sinai Medical Center, Los Angeles, CA USA; 60 Department of Human Genetics, University Würzburg, Würzburg, Germany; 61 Service Régional Oncogénétique Poitou-Charentes, CH Niort, Niort, France; 62 Department of Pathology and Laboratory Medicine, University of Kansas Medical Center, Kansas City, KS USA; 63 Department of Dermatology, Huntsman Cancer Institute, University of Utah School of Medicine, Salt Lake City, UT USA; 64 Waukesha Memorial Hospital-Pro Health Care, Waukesha, USA; 65 Molecular Genetics of Breast Cancer, German Cancer Research Center (DKFZ), Heidelberg, Germany; 66 Department of Clinical Genetics, Rigshospitalet, Copenhagen University Hospital, Copenhagen, Denmark; 67 Institute of Human Genetics, University Hospital Leipzig, Leipzig, Germany; 68 Department of Gynecology and Obstetrics, University Hospital Düsseldorf, Heinrich-Heine University Düsseldorf, Düsseldorf, Germany; 69 Center for Medical Genetics, NorthShore University HealthSystem, Evanston, IL USA; 70 The University of Chicago Pritzker School of Medicine, Chicago, IL USA; 71 N.N. Petrov Institute of Oncology, St. Petersburg, Russia; 72 Lombardi Comprehensive Cancer Center, Georgetown University, Washington, DC USA; 73 Clinical Genetics, Guy’s and St Thomas’ NHS Foundation Trust, London, UK; 74 Department of Genetics and Pathology, Pomeranian Medical University, Szczecin, Poland; 75 Independent Laboratory of Molecular Biology and Genetic Diagnostics, Pomeranian Medical University, Szczecin, Poland; 76 Parkville Familial Cancer Centre, Peter MacCallum Cancer Center, Melbourne, Victoria Australia; 77 Faculty of Medicine, Institute of Biomedical Sciences, Dept. Of Human and Medical Genetics, Vilnius University, Vilnius, Lithuania; 78 State Research Institute Centre for Innovative Medicine, Vilnius, Lithuania; 79 Department of Epidemiology & Population Health, Stanford University School of Medicine, Stanford, CA USA; 80 Department of Medicine, Division of Oncology, Stanford Cancer Institute, Stanford University School of Medicine, Stanford, CA USA; 81 David Geffen School of Medicine, Department of Obstetrics and Gynecology, University of California at Los Angeles, Los Angeles, CA USA; 82 Breast and Cancer Genetics Units, The Royal Marsden NHS Foundation Trust, London, UK; 83 Familial Cancer Service, Weatmead Hospital, Wentworthville, New South Wales Australia; 84 Department of Medical Genetics, University Medical Center, Utrecht, The Netherlands; 85 Hong Kong Hereditary Breast Cancer Family Registry, Hong Kong, China; 86 Department of Surgery, The University of Hong Kong, Hong Kong, China; 87 Department of Surgery and Cancer Genetics Center, Hong Kong Sanatorium and Hospital, Hong Kong, China; 88 Department of Population Health Sciences, Weill Cornell Medicine, New York, NY USA; 89 Unité de Prévention et d’Epidémiologie Génétique, Centre Léon Bérard, Lyon, France; 90 Department of Clinical Genetics, Aarhus University Hospital, Aarhus N, Denmark; 91 Département de Génétique, CHU de Grenoble, Grenoble, France; 92 Genetic Epidemiology of Cancer team, Inserm U900, Paris, France; 93 Institut Curie, Paris, France; 94 Mines ParisTech, Fontainebleau, France; 95 Magee-Womens Hospital, University of Pittsburgh School of Medicine, Pittsburgh, PA USA; 96 Département d’Hématologie-Oncologie Médicale, Centre Antoine Lacassagne, Nice, France; 97 Department of Medical Oncology, Erasmus MC Cancer Institute, Rotterdam, The Netherlands; 98 Department of Gynecology and Obstetrics, University of Munich, Campus Großhadern, Munich, Germany; 99 NRG Oncology, Statistics and Data Management Center, Roswell Park Comprehensive Cancer Center, Buffalo, NY USA; 100 Immunology and Molecular Oncology Unit, Veneto Institute of Oncology IOV - IRCCS, Padua, Italy; 101 Service de Génétique, Institut Curie, Paris, France; 102 Department of Clinical Genetics, Yorkshire Regional Genetics Service, Chapel Allerton Hospital, Leeds, UK; 103 Unité d’oncogénétique, Centre de Lutte Contre le Cancer, Centre Georges-François Leclerc, Dijon, France; 104 Department of Obstetrics and Gynecology, Helsinki University Hospital, University of Helsinki, Helsinki, Finland; 105 Lee Kong Chian School of Medicine, Nanyang Technological University, Singapore, Singapore; 106 Cancer Genetics Service, National Cancer Centre, Singapore, Singapore; 107 Precision Medicine, School of Clinical Sciences at Monash Health, Monash University, Clayton, Victoria Australia; 108 Department of Clinical Pathology, The University of Melbourne, Melbourne, Victoria Australia; 109 Latvian Biomedical Research and Study Centre, Riga, Latvia; 110 Clinical Genetics Service, Department of Medicine, Memorial Sloan Kettering Cancer Center, New York, NY USA; 111 Department of Molecular Genetics, National Institute of Oncology, Budapest, Hungary; 112 Center for Clinical Cancer Genetics, The University of Chicago, Chicago, IL USA; 113 Familial Cancer Clinical Unit, Human Cancer Genetics Programme, Spanish National Cancer Research Centre (CNIO) and Spanish Network on Rare Diseases (CIBERER), Madrid, Spain; 114 Institute of Medical Genetics and Human Genetics, Charité - Universitätsmedizin Berlin, corporate member of Freie Universität Berlin, Humboldt-Universität zu Berlin and Berlin Institute of Health, Berlin, Germany; 115 Department of Preventive Medicine, Seoul National University College of Medicine, Seoul, Korea; 116 Integrated Major in Innovative Medical Science, Seoul National University College of Medicine, Seoul, South Korea; 117 Cancer Research Institute, Seoul National University, Seoul, Korea; 118 Department of Genetics and Computational Biology, QIMR Berghofer Medical Research Institute, Brisbane, Queensland Australia; 119 Molecular Diagnostics, Aalborg University Hospital, Aalborg, Denmark; 120 Clinical Cancer Research Center, Aalborg University Hospital, Aalborg, Denmark; 121 Department of Clinical Medicine, Aalborg University, Aalborg, Denmark; 122 Department of Genetics, Portuguese Oncology Institute, Porto, Portugal; 123 Genome Diagnostics Program, IFOM ETS - the AIRC Institute of Molecular Oncology, Milan, Italy; 124 Unit of Molecular Bases of Genetic Risk and Genetic Testing, Department of Research, Fondazione IRCCS Istituto Nazionale dei Tumori (INT), Milan, Italy; 125 Division of Gynaecology and Obstetrics, Klinikum rechts der Isar der Technischen Universität München, Munich, Germany; 126 Clinical Genetics, Karolinska Institutet, Stockholm, Sweden; 127 Division of Gynecologic Oncology, NorthShore University HealthSystem, University of Chicago, Evanston, IL USA; 128 Department of Clinical Genetics, University Hospital of Southern Denmark, Vejle Hospital, Vejle, Denmark; 129 Department of Pathology, The Netherlands Cancer Institute - Antoni van Leeuwenhoek Hospital, Amsterdam, The Netherlands; 130 Center for Genomic Medicine, Rigshospitalet, Copenhagen, Denmark; 131 Department of Clinical Medicine, University of Copenhagen, Copenhagen, Denmark; 132 West Midlands Regional Genetics Service, Birmingham Women’s Hospital Healthcare NHS Trust, Birmingham, UK; 133 Department of Internal Medicine, Division of Medical Oncology, University of Kansas Medical Center, Westwood, KS USA; 134 Princess Anne Hospital, Southampton, UK; 135 Genomics Center, Centre Hospitalier Universitaire de Québec – Université Laval Research Center, Québec City, QC Canada; 136 Dept of OB/GYN and Comprehensive Cancer Center, Medical University of Vienna, Vienna, Austria; 137 Medical Genetics Unit, St George’s, University of London, London, UK; 138 Institute of Human Genetics, Hannover Medical School, Hannover, Germany; 139 Department of Tumour Biology, INSERM U830, Paris, France; 140 Université Paris Cité, Paris, France; 141 Institute of Human Genetics, University Hospital Heidelberg, Heidelberg, Germany; 142 Biomedical Sciences Institute (ICBAS), University of Porto, Porto, Portugal; 143 Breast Cancer Research Programme, Cancer Research Malaysia, Subang Jaya, Selangor Malaysia; 144 Department of Surgery, Faculty of Medicine, University of Malaya, Kuala Lumpur, Malaysia; 145 Department of Medicine, Magee-Womens Hospital, University of Pittsburgh School of Medicine, Pittsburgh, PA USA; 146 Program in Cancer Genetics, Departments of Human Genetics and Oncology, McGill University, Montréal, QC Canada; 147 Department of Medical Genetics, National Institute for Health Research Cambridge Biomedical Research Centre, University of Cambridge, Cambridge, UK; 148 Department of Cancer Biology and Genetics, The Ohio State University, Columbus, OH USA; 149 Department of medicine, University Of Melbourne, Melbourne, Victoria Australia; 150 South East Thames Regional Genetics Service, Guy’s and St Thomas’ NHS Foundation Trust, London, UK; 151 Department of Medical Oncology, Beth Israel Deaconess Medical Center, Boston, MA USA; 152 Department of Clinical Genetics, VU University Medical Center, Amsterdam, The Netherlands; 153 Department of Genetics, University of Pretoria, Arcadia, South Africa; 154 Centro de Investigación en Red de Enfermedades Raras (CIBERER), Madrid, Spain; 155 Fundación Pública Galega de Medicina Xenómica, Santiago de Compostela, Spain; 156 Instituto de Investigación Sanitaria de Santiago de Compostela (IDIS), Complejo Hospitalario Universitario de Santiago, SERGAS, Santiago de Compostela, Spain; 157 Division of Functional onco-genomics and genetics, Centro di Riferimento Oncologico di Aviano (CRO), IRCCS, Aviano, Italy; 158 Oxford Regional Genetics Service, Churchill Hospital, Oxford, UK; 159 Latin American School of Oncology, Tuxtla Gutiérrez, Chiapas, Mexico; 160 Radboud University Medical Center, Nijmegen, Netherlands

**Keywords:** Cancer genetics, Cancer

## Abstract

The contribution of germline copy number variants (CNVs) to risk of developing cancer in individuals with pathogenic *BRCA1* or *BRCA2* variants remains relatively unknown. We conducted the largest genome-wide analysis of CNVs in 15,342 *BRCA1* and 10,740 *BRCA2* pathogenic variant carriers. We used these results to prioritise a candidate breast cancer risk-modifier gene for laboratory analysis and biological validation. Notably, the HR for deletions in *BRCA1* suggested an elevated breast cancer risk estimate (hazard ratio (HR) = 1.21), 95% confidence interval (95% CI = 1.09–1.35) compared with non-CNV pathogenic variants. In contrast, deletions overlapping *SULT1A1* suggested a decreased breast cancer risk (HR = 0.73, 95% CI 0.59-0.91) in *BRCA1* pathogenic variant carriers. Functional analyses of *SULT1A1* showed that reduced mRNA expression in pathogenic *BRCA1* variant cells was associated with reduced cellular proliferation and reduced DNA damage after treatment with DNA damaging agents. These data provide evidence that deleterious variants in *BRCA1* plus *SULT1A1* deletions contribute to variable breast cancer risk in *BRCA1* carriers.

## Abstract (web summary)

The risk of germline copy number variants (CNVs) in BRCA1 and BRCA2 pathogenic variant carriers in breast cancer is assessed, with CNVs overlapping SULT1A1 decreasing breast cancer risk in BRCA1 carriers.

## Introduction

Women who carry pathogenic variants in *BRCA1* (OMIM 113705) and *BRCA2* (OMIM 600185) have greatly increased risk of developing breast cancer. Recent cumulative risk estimates in high-risk families for developing breast cancer by age 80 years were 72% (95% confidence interval (95% CI, 65–79%)) for *BRCA1* and 69% (95% CI, 61–77%) for *BRCA2* pathogenic. variant carriers^1^. The significant variation of age at diagnosis of breast cancer between pathogenic variant carriers suggests additional factors, such as common inherited genetic variants, influence disease penetrance^2,3^. Large genome-wide association studies, facilitated by the Consortium of Investigators of Modifiers of *BRCA1/BRCA2* (CIMBA^2,3^), have demonstrated that >60 single nucleotide polymorphisms (SNPs) or small insertions or deletions (Indels) associated with cancer risk in the general population also are associated with breast cancer risk for *BRCA1/2* pathogenic variant carriers^4–6^. Moreover, population-based breast cancer polygenic risk scores are associated with modified breast cancer risk for carriers^7,8^. However, the identified single nucleotide variant modifiers account for <10% of heritable variation in risk in *BRCA1/2* pathogenic variant carriers^5^.

Copy number variants (CNVs) cover between 5-10% of the human genome and, based on nucleotide coverage, are responsible for the majority of variation in the human genome^9,10^. CNVs exhibit substantial variability in both size and frequency and can disrupt gene function significantly by altering gene dosage, coding sequences, and gene regulation^11^. Germline CNVs overlapping the *BRCA1* and *BRCA2* gene loci are associated with the pathogenesis of breast cancer, accounting for <5% of known pathogenic variants in these genes (https://www.ncbi.nlm.nih.gov/clinvar). Multiple studies have utilised a genome-wide approach to identify associations between rare and common CNVs and the risk of developing breast cancer^12–16^.

CNVs previously have been shown to be modifiers of hereditary breast cancer risk. In a genome-wide association study (GWAS) of CNVs in 2500 *BRCA1* pathogenic variant carriers, 52 gene loci were associated (unadjusted *p* < 0.05) with breast cancer risk^14^. Although no variant reached the widely-adopted genome-wide statistical significance threshold applied for SNP-centric GWAS (*p* < 5 × 10^−8^) and the study sample size was relatively small, the specific genes disrupted by CNVs had plausible biological consequences regarding cancer development. These data suggested that CNVs are an important modifier of hereditary breast cancer risk and highlighted the need for larger and more comprehensive CNV studies.

In this study, we conducted genome-wide CNV analyses of 15,342 *BRCA1* and 10,740 *BRCA2* pathogenic variant carriers using genotype data generated by the OncoArray Network^17^. We applied in silico and in vitro analyses to characterise a novel risk association between deletions overlapping *SULT1A1* (OMIM 171150) and decreased breast cancer risk for *BRCA1* pathogenic variant carriers.

## Results

### Copy number variants

857,647 CNVs (327,530 deletions and 530,117 duplications) were called in study participants, of which 374,210 CNVs (43.6%; 136,534 deletions and 237,676 duplications) overlapped at least one of 16,395 different gene regions. On average, each genome carried 14.4 CNVs (range = 0–63) that overlapped an average of 21.7 genes (range = 0–236). On average, duplications were detected nearly twice as often as deletions (9.1 (range = 0–55) versus 5.2 (range = 0–58), respectively) and affected twice as many gene regions (14.6 (range = 0–220) versus 7.0 (range = 0–207), respectively).

### Evaluation of CNV calling

The sensitivity and specificity of our CNV calling was assessed by comparing diagnostically identified *BRCA1* and *BRCA2* CNVs to the CNVs called by our analysis. In our cohort, 1,138 *BRCA1* and 166 *BRCA2* diagnostically identified CNVs overlapped five or more probes and passed our variant filtering; we detected 678 and 155 for *BRCA1* and *BRCA2*, respectively. Furthermore, our genome-wide analysis called 851 *BRCA1* and 183 *BRCA2* CNVs, of which 151 and 28 CNVs were not supported by diagnostic testing of *BRCA1* and *BRCA2*, respectively. Together, our CNV calling achieved an 82.2% and 84.7% detection specificity and 59.6% and 92.8% detection sensitivity for CNVs in *BRCA1* and *BRCA2*, respectively.

Separate analysis of deletions and duplications found that PennCNV performed better with deletion calling verses duplication calling (Supplementary Data 1). The sensitivity of calling *BRCA1* and *BRCA2* deletions were 84.1% and 91.3%, respectively, while the sensitivity of calling *BRCA1* and *BRCA2* duplications were 68.6% and 39.1%, respectively. Similarly, the specificity of calling *BRCA1* and *BRCA2* deletions was 70.4% and 94.8% and duplications was 25.0% and 60.0%, respectively. A review of the diagnostic CNVs not called by PennCNV found that the majority of uncalled CNVs overlapped were the same variants. For example, 54.1% of *BRCA1* deletions not detected by PennCNV were the same variant (c.5333-36_5406 + 400del510), which was only successfully called by PennCNV for 1.4% of the variant carriers.

Genome-wide CNV calling was assessed further for three cases using whole-genome sequencing (WGS). In the three whole-genome sequenced cases, 4444 (Case 1), 4540 (Case 2), and 4545 (Case 3) CNV calls passed confidence filtering, of which 1884 (Case 1), 1981 (Case 2), and 1940 (Case 3) overlapped gene regions, respectively (Supplementary Data 2). A total of 10 of 14 (71.4%—Case 1), 23 of 42 (54.8%—Case 2), and 13 of 19 (68.4%—Case 3) of PennCNV calls were supported by a CNV called in the WGS data (Supplementary Data 3). Of the CNVs not supported by WGS, 4 of 4 (100%—Case 1), 12 of 19 (63.2%—Case 2), and 6 of 6 (100%—Case 3), CNVs were supported by a previously-published CNV map^10^. All duplications that were not supported by WGS were supported by the CNV map while approximately half the deletions were not (Supplementary Data 4). Each of the three cases also carried a diagnostically identified pathogenic *BRCA1* deletion that was called by WGS CNV calling. Together, these data provide confidence that >80% of deletions and duplications called by PennCNV in this study cohort appear to be true calls.

### Prioritization of candidate breast cancer CNV risk loci

To prioritise genes for in silico and functional analyses, we selected candidate gene loci with *p* < 0.01 from retrospective likelihood analysis, effectively restricting hazard ratios to >1.25 and <0.75 (Supplementary Data 5–8). Putative CNVs at 31 gene regions passed this threshold. For 16 of these 31 regions, the proportion of unique CNVs represented in a published human CNV map^10^ was <95%. Although none of the CNV regions passed significance thresholds when adjusted for multiple hypothesis testing (See Methods; deletions in *BRCA1* carriers—*p* ≤ 8 × 10^−6^; duplications in *BRCA1* carriers—*p* ≤ 5 × 10^−6^; deletions in *BRCA2* carriers—*p* ≤ 1 × 10^−5^; and duplications in *BRCA2* carriers—*p* ≤ 6 × 10^−6^), we used these results to prioritise a candidate risk-modifier gene for laboratory analysis and biological validation.

Deletions overlapping *BRCA1* increased breast cancer risk (hazard ratio (HR) = 1.29, 95%CI = 1.13–1.49, *p* = 1.98 × 10^−4^) (Supplementary Data 5) for *BRCA1* pathogenic variant carriers. This result was explored further as the analysis did not directly compare the effect of *BRCA1* deletions and *BRCA1* non-deletion pathogenic variants. Clinically diagnosed variants for *BRCA1* and *BRCA2* carriers were categorised by type (deletions, duplications, and small variants [i.e. nonsense, missense, frame shift, Indel, and splice site]). Assessing the HRs for CNV *versus* non-CNV pathogenic variants, separately for *BRCA1* and *BRCA2* suggested elevated breast cancer risk for *BRCA1* deletions (HR = 1.21, 95%CI = 1.09–1.35) but not *BRCA2* deletions (Table 1, Supplementary Data 9). These results remained similar after excluding missense variant carriers from the analysis (Supplementary Data 9). Estimated HRs were elevated for duplications versus non-duplication pathogenic variants (deletions were excluded) for *BRCA1* duplication carriers (HR = 1.21, 95%CI = 0.99–1.48; *p* = 0.066), and *BRCA2* duplication carriers (HR = 1.52, 95%CI = 0.61–3.77, *p* = 0.39); however, results for *BRCA2* were less definitive given the smaller sample size and wide confidence intervals.

**Table 1:** Breast cancer hazard ratio estimates using a single model comparing other *BRCA1/2* pathogenic variants versus (1) deletions, and (2) duplications^a^.

	Mutation type	Unaffected	Affected	HR	95%CI		*p*
***BRCA1*** **carriers**	Other	8669	8483	1.00	[reference]		
	Deletions	636	789	1.21	1.09	1.35	4.35E-04
	Duplications	153	192	1.21	0.99	1.48	6.60E-02
***BRCA2*** **carriers**	Other	5913	6204	1.00	[reference]		
	Deletions	85	111	1.11	0.79	1.54	5.54E-01
	Duplications	6	16	1.52	0.61	3.77	3.68E-01

^a^ Weighted cohort models fitted separately for *BRCA1* and *BRCA2* carriers. Weights were calculated assuming *BRCA1/2* carrier breast cancer incidences from most recent age cohort in Antoniou et al., (2008). Models were stratified by country and Ashkenazi Jewish ancestry, and adjusted for birth cohort and genotyping array. Cluster robust variances were estimated using families as clusters.

*HR* hazard ratio, 95%*CI* 95% confidence interval; *p*-value.

Putative duplications overlapping the breast cancer tumour suppressor gene *STK11* suggested decreased risk of breast cancer in our study for both *BRCA1* carriers (HR = 0.49, 95%CI 0.29–0.81, *p* = 5.4 × 10^−3^) and *BRCA2* carriers (HR = 0.44, 95%CI 0.22–0.88, *p* = 9.2 × 10^−3^). Putative deletions overlapping *TERT* and duplications overlapping *LSP1*, two loci previously shown to be associated with breast cancer risk for *BRCA1* (*TERT* locus) and *BRCA2* (*TERT* and *LSP1* loci) pathogenic variant carriers from SNP-based studies^18,19^, suggested increased risk (HR = 1.92, 95%CI = 1.06–3.46, *p* = 6.0 × 10^−3^) and decreased risk (HR = 0.13, 95%CI = 0.04–0.45, *p* = 3.3 × 10^−3^) breast cancer risk for *BRCA2* pathogenic variant carriers in this study, respectively. However, analysis of *TERT* and *LSP1* CNVs using TaqMan assays with available DNA showed only 25% (1/4) of predicted deletions overlapping *TERT* and 50% (1/2) predicted duplications overlapping *LSP1* were successfully validated (Table 2, Supplementary Fig. 1). These results are consistent with the observation that predicted CNVs overlapping *LSP1* and *TERT* were not found in a published genomic map of human CNVs^10^.

**Table 2:** Putative copy number variants assessed by TaqMan assays.

Gene symbol	CNV type	Variant carrier	Assay ID	Overlap CNV map^1^	Proportion validated
***SULT1A1***	Deletion	*BRCA1*	1	Yes	100% (8/8)
***TERT***	Deletion	*BRCA2*	1	No	0% (0/1)
			2	No	0% (0/1)
			3	No	33% (1/3)
***LSP1***	Duplication	*BRCA2*	1	No	50% (1/2)

*CNV* copy number variant. ^1^Zarrei et al (2015) stringent CNV map.

### Identification of *SULT1A1* as a candidate modifier gene

CNV loci suggested to modify breast cancer risk estimates in *BRCA1/2* pathogenic variant carriers, were examined to identify a candidate gene for functional characterisation using in silico and in vitro assays. *SULT1A1* (sulfotransferase 1A1) was selected as a novel candidate modifier based on potential biological mechanisms of action and because overlapping CNVs had a population frequency above 1%.

**Fig. 1:**
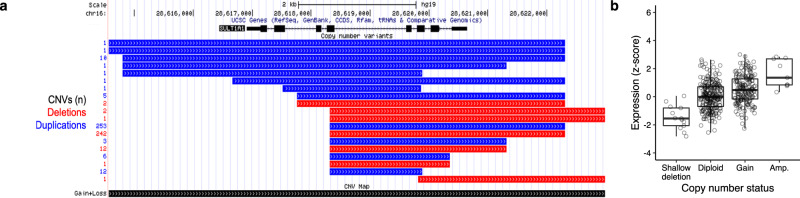
Characterisation of SULT1A1. **a** Genomic viewer (UCSC) of the SULT1A1 gene locus with copy number variants; deletion (red) and duplication (blue). **b** Dosage effect in breast tumours with SULT1A1 copy number variants. CNVs copy number variants.

In our study, CNV deletions overlapping *SULT1A1* were identified in 1.7% of *BRCA1* pathogenic variant carriers and they suggested a decreased breast cancer HR (HR = 0.73, 95%CI = 0.59–0.91, *p* = 9.1 × 10^−3^). Deletions overlapping all eight *SULT1A1* exons of the reference transcript were validated in all eight available DNA samples using both TaqMan assays and multiplex ligation-dependent probe amplification (MLPA; Supplementary Fig. 2), and were identified in the CNV map published by Zarrei et al (Supplementary Fig. 1c). Furthermore, CNVs involving the *SULT1A1* gene locus had an expression dosage effect in breast tumours (Fig. 1).

### Characterisation of *SULT1A1* knockdown in *BRCA1*^+/−^ cells

**Fig. 2:**
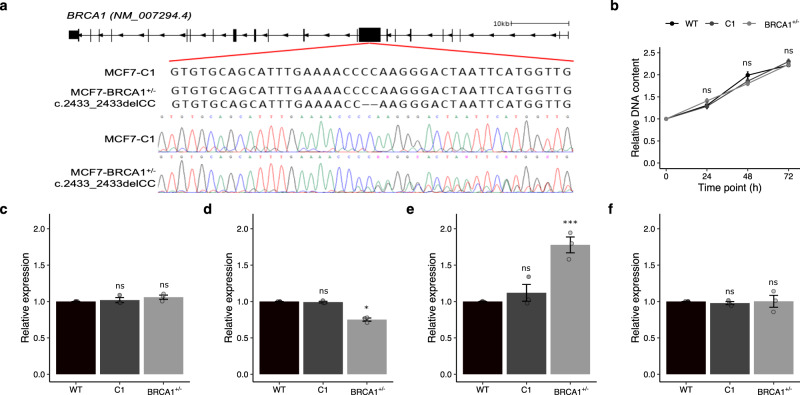
Characterisation of MCF7–WT, and isogenic MCF7–C1, and MCF7–BRCA1^+/−^ cell lines. **a** Sequence of heterozygous pathogenic BRCA1 c.2432_2433del variant introduced by CRISPR-Cas9. **b** Relative proliferation of MCF7–WT and clonally expanded CRISPR-Cas9 MCF7–C1 and MCF7–BRCA1^+/−^ cells for 72 h post seeding. Relative expression of **c** BRCA1, **d** ESR1, **e** CYP1A1, and **f** SULT1A1 for MCF7–WT, MCF7–C1 and MCF7–BRCA1^+/−^ cells. 4-OHE2 4-hydroxyestradiol, MMC Mitomycin C, Error bars = standard error of the mean; ns = *p* > 0.05; *p < 0.05; **p < 0.01; ***p < 0.001; *n* = 3 independent biological replicates; unpaired two-sided *t*-test.

To model the effect of *SULT1A1* deletions in *BRCA1* carriers, a pair of isogenic MCF7 breast cell lines with and without a pathogenic variant (*BRCA1* c.2432_2433del) were created using CRISPR-Cas9 (Fig. 2a). A comparison of the isogenic control (MCF7–C1) and pathogenic variant carrying cells (MCF7–*BRCA1*^*+/*−^) with the parent MCF7 cells (MCF7–WT) showed no significant difference in cell proliferation (Fig. 2b, Supplementary Data 10). There were also no differences in the relative expression of BRCA1 mRNA between the isogenic MCF7–C1 and MCF7–BRCA1^+/−^ lines and the parent MCF7–WT line (Fig. 2c, Supplementary Data 10). However, the MCF7–BRCA1^+/−^ cells showed a significant 25% (*p* = 4.44 × 10^−4^) reduction in ESR1 expression (Fig. 2d, Supplementary Data 10) and a significant 78% (*p* = 2.03 × 10^−3^) increase in CYP1A1 expression (Fig. 2e, Supplementary Data 10), consistent with breast cells with a pathogenic *BRCA1* variant^20^. There was no significant difference in *SULT1A1* mRNA expression between the MCF7–WT and the isogenic MCF7–C1 and MCF7–*BRCA1*^+/−^ cells (Fig. 2f, Supplementary Data 10).

**Fig. 3:**
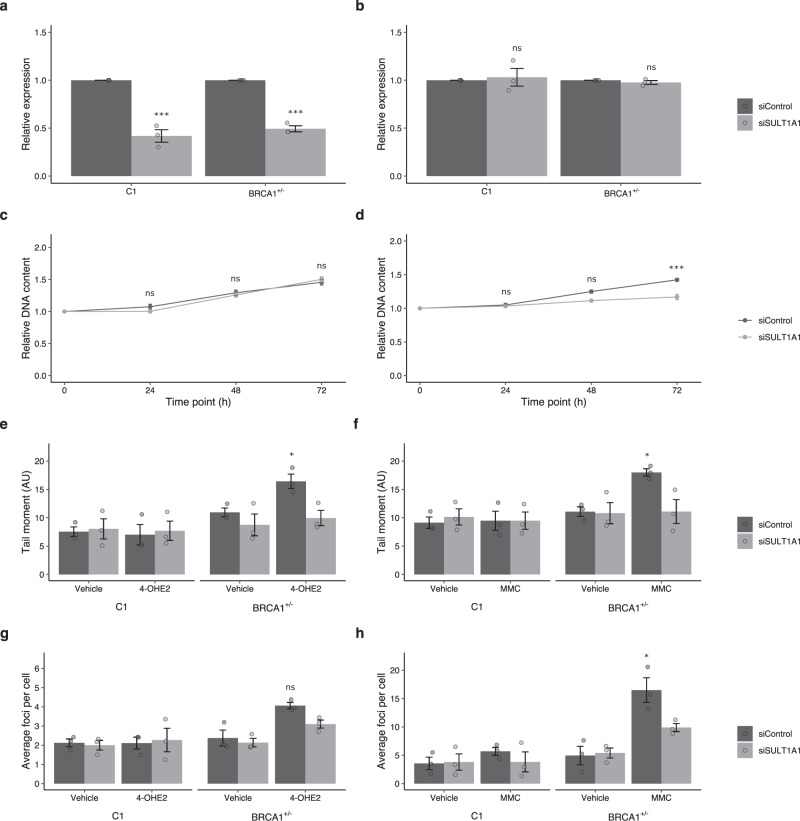
siRNA knockdown of SULT1A1 reduces proliferation and DNA damage of MCF7–BRCA1^+/−^ cells but not MCF7–C1 cells. Expression of (**a**) SULT1A1 and (**b**) BRCA1 72 h post transfection. Relative DNA content of transfected (**c**) MCF7–C1 and (**d**) MCF7–BRCA1^+/−^ cells 72 h post transfection. Quantification of transfected DNA damage using the comet assay (**e**, **f**) and ϒ-H2AX/53BP1 foci quantification (**g**, **h**) for MCF7–C1 and MCF7–BRCA1^+/−^ cells, with and without siSULT1A1 transfection, 21 h post treatment with 1 μm 4-OHE2 (**e** and **g**) or 10 μm MMC (**f** and **h**). Significance of differences in relative expression and DNA content was determined by unpaired two-sided *t*-test. Gene expression and DNA content of siSULT1A1 transfected cells normalised to siControl transfected cells. Differences in DNA damage were determined by two-way analysis of variance. Example images of comet and ϒ-H2AX/53BP1 foci shown in Supplementary Figs. 3–6. 4-OHE2 = 4-hydroxyestradiol; MMC = Mitomycin C; Error bars = standard error of the mean; ns = *p* > 0.05; *p < 0.05; **p < 0.01; ***p < 0.001; *n* = 3 independent biological replicates.

siRNA was used to transiently reduce the relative expression of *SULT1A1* mRNA in the isogenic MCF7–C1 and MCF7–*BRCA1*^+/−^ lines. Compared with the non-targeting siRNA control, the relative expression of *SULT1A1* was approximately half in both isogenic lines 72 h after transfection targeting *SULT1A1* (Fig. 3a, Supplementary Data 11). As deletions overlapping *SULT1A1* were suggested to decrease breast cancer risk in *BRCA1* pathogenic variant carriers, the relative expression of *BRCA1* was quantified to assess if the *SULT1A1* knockdown affected its regulation (Fig. 3b, Supplementary Data 11). However, there was no significant change in the *BRCA1* expression for the *SULT1A1* knockdown cells compared with the transfection control for either of the MCF7–C1 or the MCF7–*BRCA1*^+/−^ lines.

The proliferation of the transfected MCF7–C1 and MCF7–*BRCA1*^+/−^ cells was assessed by measuring relative DNA content. Knockdown of *SULT1A1* expression did not alter the proliferation of the MCF7–C1 compared with the transfection control (Fig. 3c, Supplementary Data 11). However, when *SULT1A1* expression was reduced in MCF7–BRCA1^+/−^ cells, there was a 14% (*p* = 1.17 × 10^−2^) decrease in proliferation compared with the transfection control after 72 h of growth (Fig. 3d, Supplementary Data 11).

Because BRCA1-deficient cells are hypersensitive to DNA damaging agents and have an impaired DNA damage repair response^20,21^, we investigated whether knockdown of *SULT1A1* expression altered the amount of damage caused by the DNA damaging agents 4-hydroxyestradiol (4-OHE2) and Mitomycin C (MMC) using the well-established comet and γH2AX and 53BP1 immunostaining assays. Analysis using comet assays, showed that treatment with 4-OHE2 (*F*(1, 8) = 5.79, *p* = 4.28 × 10^−2^; Fig. 3e, Supplementary Data 11) and MMC (*F*(1, 8) = 5.73, *p* = 4.36 × 10^−2^; Fig. 3f, Supplementary Data 11) increased comet tail moment length in MCF7–BRCA1^+/−^ cells. There also was evidence that transfection with siSULT1A1 reduced the average comet tail moment length for both 4-OHE2 (*F*(1, 8) = 9.76, *p* = 1.41 × 10^−2^; Fig. 3e, Supplementary Data 11) and MMC (*F*(1, 8) = 5.69, *p* = 4.43 × 10^−2^; Fig. 3f, Supplementary Data 11) treated MCF7–BRCA1^+/−^ cells. Assessing the effect of *SULT1A1* knockdown in MCF7–BRCA1^+/−^ and MCF7–C1 cells using the γ-H2AX and 53BP1 immunostaining assay gave rise to analogous results (Fig. 3e–h). Although there was evidence that treatment with 4-OHE2 increased the average number of co-localised DNA damage foci (*F*(1, 8) = 24.36, *p* = 1.14 × 10^−3^; Fig. 3g, Supplementary Data 11) in MCF7–BRCA1^+/−^ cells, the reduction in the number of foci caused by the siSULT1A1 transfection did not reach statistical significance (*F*(1, 8) = 5.02, *p* = 5.5 × 10^−2^). Additionally, for the MMC treated MCF7–BRCA1^+/−^ cells there was a significant interaction between the siSULT1A1 transfection and MMC treatment (*F*(1, 8) = 5.80, *p* = 4.3 × 10^−2^; Fig. 3h, Supplementary Data 11). This effect was significant for MMC treated MCF7–BRCA1^+/−^ cells (*F*(1, 4) = 8.44, *p* = 4.4 × 10^−2^) but not the vehicle control (*F*(1, 4) = 0.06, *p* = 8.19 × 10^−1^). There was no evidence that siRNA transfection or drug treatments affected the average comet tail moment or the number of γ-H2AX and 53BP1 foci for the MCF7–C1 cells. Example images of comet and γ-H2AX and 53BP1 immunostaining assay are shown in Supplementary Figs. 3–6.

## Discussion

Germline CNVs are an important source of genetic variation that have previously been understudied in relation to breast and ovarian cancer risk. Here, we have conducted the largest and most comprehensive genome-wide association study of CNVs and breast cancer risk for *BRCA1* and *BRCA2* pathogenic variant carriers. We identified putative CNVs in up to 31 putative gene regions that were associated (unadjusted *P* < 0.01) with breast cancer risk for *BRCA1/2* pathogenic variant carriers, with CNVs at 15 of these regions present in a human CNV map^10^. Although none of the CNV regions passed significance thresholds when adjusted for multiple hypothesis testing, we used these results to prioritise a candidate risk-modifier gene for laboratory analysis and biological validation. Consistent with observations from the human CNV map, we validated positive CNV calls overlapping the *SULT1A1* gene, and revealed false positive CNV calls at two candidate modifier gene regions (*LSP1* and *TERT*). CNV deletions overlapping the lead candidate modifier *SULT1A1* showed decreased breast cancer risk in *BRCA1* pathogenic variant carriers. In silico analysis of *SULT1A1* suggested that deletions overlapping this gene leads to reduced expression. In vitro analyses showed that reduced *SULT1A1* expression in cells carrying a heterozygous *BRCA1* pathogenic variant led to reduced cellular proliferation and reduced DNA damage after treatment with DNA damaging agents.

Both SNP and CNV variants at the *SULT1A1* locus have previously been shown to be associated with SULT1A1 enzymatic activity. The common *SULT1A1* p.(Arg213His) (rs9282861) polymorphism leading to the SULT1A1*2 variant has been examined in a series of functional and association studies. SULT1A1*2 has a two-fold lower catalytic activity and stability than its high-activity p.Arg213 counterpart (SULT1A1*1), and has been associated with increased cancer risk in multiple tissue types^22–25^. Studies examining the association between the rs9282861 polymorphism and breast cancer risk have yielded inconsistent results showing an increase in risk in some studies^26–29^ and no association in others^30–32^. The OncoArray probe for rs9282861 failed quality control, therefore no genotype data was available. Interestingly, the rs200802208 Indel located near *SULT1A1* was imputed from these arrays and analyses showed that the del allele was associated with decreased risk of breast cancer in *BRCA1* pathogenic variant carriers (HR = 0.48, 95%CI 0.29–0.79, *p* = 4.3 × 10^−3^)^5^. rs9282861 is not in 1000 G reference panel, therefore it is unknown whether this SNP exists in linkage disequilibrium with rs200802208. CNVs overlapping *SULT1A1* are strongly associated with SULT1A1 activity and explain more of the observed in vitro variability in SULT1A1 activity than SNPs, with activity proportional to *SULT1A1* copy number^33–36^. Individuals who are homozygous null for *SULT1A1* do not present with any overt phenotypes^37^. This finding corresponds with results from phenotypic analyses of mouse *SULT1A1* knockouts which are viable, and which also lack any outward phenotype^38,39^. However, the absence of functional *SULT1A1* enzyme in mouse knockouts has been reported to reduce the number of DNA adducts caused by DNA damaging agents that are converted to mutagenic metabolites by SULT1A1^39^.

The mechanism by which CNV deletions overlapping *SULT1A1* were associated with lower *BRCA1*-associated breast cancer risk may be linked to the production of potentially toxic catechol oestrogens by the Cytochromes P450 (CYP) enzymes. SULT1A1 is an important SULT isoform that is expressed widely in human tissues and plays an important role in the metabolism, bioactivation, and detoxification of carcinogens, medications, and steroid hormones^33,34^. *SULT1A1* has established germline common CNVs and SNPs that are known to alter its activity^40^. *SULT1A1* is most abundantly expressed in the liver, but is also expressed in the brain, breast, intestine, and endometrium^41–44^. *SULT1A1* expression is related to disease state, with plentiful expression in most breast tumours^42,45^. In normal breast cells, BRCA1 regulates oestrogen metabolism and metabolite-mediated DNA damage by repressing transcription of the oestrogen-metabolising enzyme CYP1A1^20,46^. However, levels of CYP1A1 are higher in breast cells lacking *BRCA1* function and promote the formation of the carcinogenic 2-hydroxyestradiol (2-OHE2)^20^. Further metabolism of 2-OHE2 by catechol O-methyltransferase (COMT) is thought to have a risk reducing effect by catalysing the formation of 2-methoxyestradiol (2-MeOE2), a metabolite which interacts with the tubulin colchicine-binding site during polymerisation and which has anticarcinogenic effects by suppressing cell proliferation. In turn, SULT1A1 is an efficient catalyst of 2-MeOE2 sulfation producing 2-MeOE2-3S, a sulfate conjugate with diminished activity^33,47^. It is possible that the decreased risk associated with *SULT1A1* deletions for *BRCA1* pathogenic variant carriers and the decrease in cell proliferation and amount of DNA damage for MCF7–BRCA1^+/−^ with a SULT1A1 knockdown cells may both be linked to 2-MeOE2 abundance. That is, reduced SULT1A1 activity promotes the accumulation of 2-MeOE2 and slows the proliferation of breast cells with unbalanced E2 metabolism. Indeed, the SULT1A1 substrate, 2-MeOE2, has previously been proposed as a potential preventative agent for breast cancer^48^. A similar relationship between CYP1A1 and SULT1A1 activity and reduced breast cancer risk has been demonstrated previously. In a study of pairwise combinations of oestrogen metabolism alleles and breast cancer risk, the SULT1A1*2 genotype was assessed in combination with a *CYP1A1* missense variant (CYP1A1*2 *C*) that has increased inducibility to produce catechol oestrogens^49^. For European-Americans, carrying the CYP1A1*2 *C* genotype was associated with increased breast cancer risk (odds ratio (OR) = 1.71, 95%CI = 1.09–2.67). However, carrying the CYP1A1*2 *C* allele in combination with a SULT1A1*2 allele was strongly protective against developing breast cancer (OR = 0.14, 95%CI = 0.04–0.56) compared with women carrying only the CYP1A1*2 *C* allele. There was no association between the CYP1A1*2 *C* (rs1048943) polymorphism and breast cancer risk in *BRCA1* pathogenic variant carriers^5^. These results further suggest that the balance between the generation of catecholestrogens and catecholestrogen sulfation may be an important mechanism for modulating breast cancer risk and worthy of future investigation.

Our study provides strong evidence that deletions overlapping *BRCA1* are associated with a 1.21-fold higher risk of developing breast cancer. Large deletions in *BRCA1* have previously been shown to be associated with an increased risk of breast cancer risk (OR = 1.42) compared with carriers of *BRCA1* pathogenic single nucleotide variants or Indels^50^. Similarly, a series of studies have reported a higher incidence of CNVs in both *BRCA1* and *BRCA2* when cases have a family history that includes high-risk features, such as early-onset disease^51,52^. Although a mechanism that explains the higher risk for *BRCA1* deletion carriers is unclear, one possible explanation is that large genomic variants disrupt key BRCA1 domains or cause nonsense-mediated mRNA decay, whereas some single nucleotide variants in *BRCA1* avoid nonsense-mediated decay and retain partial function. For example, the p.(Arg1699Gln) variant in *BRCA1* produces a protein with ambiguous behaviour in a variety of functional assays and is also associated with an intermediate risk^53,54^. Furthermore, variants in both *BRCA1* and *BRCA2* are proposed to have variant-specific risks, that coincide with known or hypothesised functional domains and vary by variant type and location^1,55^. Our results support the hypothesis that breast cancer risk for women carrying large deletions in *BRCA1* is greater than those pathogenic single nucleotide variants or Indels, which may have implications for clinical risk assessment and management of CNV carriers.

Despite this being the largest sample size of *BRCA1* and *BRCA2* pathogenic variant carriers available to date, the low frequency of CNVs results in limited power for detecting significant associations after adjusting for multiple comparisons. As a result, a nominal screening threshold of 0.01 was used which is arbitrary and is therefore a limitation of the study. Nevertheless, this is the largest extant dataset available for examining genetic modifiers of *BRCA1* and *BRCA2* related risk. While larger studies, such as the new Confluence project (https://dceg.cancer.gov/research/cancer-types/breast-cancer/confluence-project), may lead to improved statistical power to detect CNV associations, evaluating uncommon genetic variation such as CNVs that overlap *SULT1A1* and other potential modifier genes in *BRCA1/2* pathogenic variant carriers will remain a challenge. Furthermore, CNV calling algorithms have limitations which lead to false CNV calls, thus highlighting the importance of using ancillary data to prioritise regions for downstream analyses. Here we show that functional analysis of a candidate modifier gene using a model cell line is able to provide additional evidence that *SULT1A1* deletions lead to reduced risk of breast cancer in *BRCA1* pathogenic variant carriers. If verified, future therapeutic intervention studies targeting SULT1A1 in *BRCA1* pathogenic variant carriers may lead to new medical options for reducing breast cancer risk.

In conclusion, our study provides evidence that CNVs contribute to the variability in breast cancer risk among *BRCA1* and *BRCA2* pathogenic variant carriers. Characterising pathogenic variant type in *BRCA1*, and future screening for deletions overlapping *SULT1A1*, may produce variables to be incorporated with other modifying factors to develop a more comprehensive model of breast cancer risk. For example, integrating these genetic data into the CanRisk Web Tool (https://www.canrisk.org/)^56^ along with family history, lifestyle/hormonal risk factors, common genetic susceptibility variants, and mammographic density, may further improve breast cancer risk predictions. Such a model may better inform patient decisions regarding breast cancer risk management.

## Methods

### Study cohort

Female *BRCA1* and *BRCA2* pathogenic variant carriers were from study centres across North America, Europe, and Australia participating in CIMBA (Supplementary Data 12), as reported previously^4,5^. Eligibility criteria for study participants included: (1) female carriers of *BRCA1* or *BRCA2* pathogenic variants; and (2) minimum 18 years of age at recruitment. A complete list of *BRCA1* and *BRCA2* pathogenic variants are deposited in the ClinVar database (https://www.ncbi.nlm.nih.gov/clinvar/submitters/505954/) and a filtered list of those in participants that were analysed in this study (post-quality control) is shown in Supplementary Data 13. There were 7725 (50.4%) *BRCA1* and 5488 (51.1%) *BRCA2* pathogenic variant carriers diagnosed with breast cancer (Supplementary Data 14). All participants were recruited for research studies using ethically approved protocols at host institutions.

### CNV detection and quality control

DNA samples were genotyped using the OncoArray-500k BeadChip (Illumina) with 533,631 probes, and standard sample quality control exclusions were performed as previously described for the SNP genotype analysis^17^. GenomeStudio (Illumina) was used to export Log R Ratios (LRRs) and B allele frequencies (BAFs) for each sample as previous described^14^. A principal components adjustment (PCA) was run on the LRR to remove noise using the bigpca package (V1.1)^57^ in the statistical platform R (V3.5.2)^58^. CNV calls were generated using PennCNV^59^. Probes that failed to cluster using Illumina’s Gentrain algorithm (*n* = 4857) and probes on the Y chromosome were removed from these results. Neighbouring CNVs with a gap of <20% of the total length of the combined CNVs, were merged using the PennCNV clean_cnv.pl script. For the current study we determined genetic ancestry using a principal components approach described elsewhere^5^. A total of 15679 *BRCA1* and 10981 *BRCA2* pathogenic variant carriers of European ancestry were assessed.

The study cohort was filtered to remove samples that failed study requirements or quality controls (Supplementary Fig. 7). Samples were removed if they met criteria listed in Supplementary Fig. 7, or if they met the following criteria: PennCNV measures of LRR standard deviation (s.d.) > 0.28, BAF drift > 0.01, waviness factor deviating from 0 by >0.05; LRR outliers > 0.1, BAF s.d. ≥ 0.2, LRR s.d. ≥ 0.4. Additionally, samples with >100 CNVs were excluded. To reduce false positive calls, only copy number variants called by five or more probes were retained for analysis. A total of 857,647 CNVs carried by 15,342 *BRCA1* and 10,740 *BRCA2* pathogenic variant carriers passed quality control steps and were assessed.

### Defining gene-centric CNVs

To identify genomic loci that influence breast cancer risk for *BRCA1* and *BRCA2* pathogenic variant carriers, a non-redundant gene-centric approach was used. Gene regions were derived from the University of California, Santa Cruz (UCSC) GRCh37/Hg19 gene track (updated: 14 June 2013) and were restricted to chromosomes 1–22, and chromosome X. In total, 30,336 gene regions with 27,038 unique gene symbols were derived and used in our analysis. CNVs that overlapped a gene region by one or more base pairs were identified in a genome-wide scan in R (V3.5.3) using the GenomicRanges package (V1.4)^60^. Overall, 374,210 CNVs overlapped one or more of 16,395 unique gene regions and were retained for statistical analysis.

### Breast cancer risk association analysis

The association analyses between breast cancer risk and copy number deletions and duplications were conducted separately for *BRCA1* and *BRCA2* pathogenic variant carriers. Study participants were followed from birth until the age at first breast cancer diagnosis, age at ovarian cancer diagnosis or bilateral prophylactic mastectomy (whichever occurred first), or at the age at last observation. Only those diagnosed with breast cancer were considered to be affected. Pathogenic variant carriers with ovarian cancer were considered unaffected, and censored at ovarian cancer diagnosis.

*BRCA1* and *BRCA2* pathogenic variant carriers were sampled non-randomly with respect to their disease status. Therefore, to evaluate associations between deletions and duplications and breast cancer risk, we analysed these data using a kinship adjusted score test based on the retrospective likelihood of observing the CNV conditional on the observed phenotype to account for the non-random ascertainment^61^. This model is stratified by country and Ashkenazi Jewish ancestry but is unable to adjust for covariates. An approximation method yields HR and 95%CI estimates based on this score test^61^. Instances in which a non-overlapping deletion and duplication was called in the same gene region were excluded, however this occurrence was relatively uncommon (<1% of participants were removed after the analysis of 99.3% of gene regions). Retrospective likelihood analysis of variants was performed using R (V3.3.1) and bespoke software (available on request). Conservative significance thresholds were based on the number of effective tests in this gene-centric CNV study. After excluding gene regions with no overlapping CNVs, thresholds were as follows: deletions in *BRCA1* carriers—*p* ≤ 0.05/6551 = 8 × 10^−6^; duplications in *BRCA1* carriers—*p* ≤ 0.05/10240 = 5 × 10^−6^; deletions in *BRCA2* carriers—*p* ≤ 0.05/5094 = 1 × 10^−5^; and duplications in *BRCA2* carriers—*p* ≤ 0.05/8469 = 6 × 10^−6^. Hypervariable regions of the genome that are prone to CNV calling errors, including the human leucocyte antigens, immunoglobulin superfamily, and olfactory receptor genes were excluded from the final gene lists.

Models to estimate the associations (HRs) of deletions and duplications simultaneously took the form of a weighted cohort analysis^61,62^. This method assigns different weights to unaffected and affected carriers depending on their age at diagnosis/censoring such that the weighted cohort mimics a true cohort. Weights were calculated using the most recent birth cohort incidence estimates from Antoniou et al.^18^. These models were stratified by country and Ashkenazi Jewish ancestry, and further adjusted for genotyping array (iCOGS or OncoArray) birth cohort (<1920, 1920-29, 1930-39, 1940-49, ≥1950). To account for relatedness, cluster robust variances were estimated using unique family IDs as clusters.

### CNV validation

Gene loci found associated with risk were reviewed to identify CNVs for validation using orthogonal technologies. CNVs were prioritised for validation if one or more DNA samples were available, the gene locus was associated with risk (unadjusted *p*-value < 0.01), and if they overlapped a gene region that had been previously associated with breast cancer risk. Copy number assessment was carried out using TaqMan assays for five different copy number variable regions in three different genes, including one gene (*SULT1A1*) that also was assayed with MLPA. Custom primer and probe sequences and pre-designed assays from Life Technologies used to validate CNVs are listed in Supplementary Data 15. *SULT1A1* MLPA was performed using the *SULT1A1* MLPA kit (C1-0217; MRC-Holland) and analysed using Coffalyser software (v9.4; MRC-Holland), as per the manufacturer’s instructions.

Three samples also were evaluated using WGS to assess the genome-wide accuracy of CNV calling. Libraries were prepared for whole-genome sequencing using the KAPA Hyper PCR Free Library preparation kit (V2.1) and 2 × 150 bp paired end sequenced on the Illumina HiSeqX platform (Kinghorn Centre for Clinical Genomics, Australia). Genomic data were processed using a modified version of the GATK best practise guidelines. Cases were sequenced to an average of 30-fold depth and CNVs were called using Lumpy and CNVnator (Supplementary Data 16). Briefly, FastQ files were generated and adaptors trimmed using Illumina’s Bcl2fastq (V2.16). Reads were aligned to the b37d5 (1000 Genomes Project GRCh37 plus decoy) reference genome using BWA-mem (V0.7.10-r789)^63^, followed by Novosort (V1.03.01) to create coordinated-sorted duplicate marked files. GATK (V3.3) Indel realignment and base quality recalibration were used to create analysis ready reads^64^. Single nucleotide variants and short insertions and deletions were joint‐called using GATK HaplotypeCaller in gVCF mode with variant quality score recalibration. Structural variants, including CNVs, were distinguished from split reads and discordant pairs using lumpy (V0.2.13)^65^ and read depth differences using CNVnator (V0.3.3)^66^.

### Dosage effect analysis

Expression and copy number data from the Breast Invasive Carcinoma^67^ datasets were downloaded using cBioPortal^68^. mRNA expression was calculated as a Z-score from all genes and putative copy number alterations were calculated using GISTIC.

### Cell culture

The MCF7 breast cancer cell line was purchased from the American Type Culture Collection (ATCC) and maintained in Dulbecco′s Modified Eagle′s Medium (DMEM) supplemented with 10% (v/v) foetal bovine serum (FBS). Cells were cultured in a humidified atmosphere of 5% CO_2_ at 37 °C, and routinely passaged every 3–4 days. Cells were used up to a maximum of 30 passages.

### Development of MCF7–*BRCA1*^+/−^ cell line

MCF7 cells underwent CRISPR-Cas9 editing to create isogenic cells with and without a heterozygous *BRCA1* frameshift variant resulting in premature truncation of the protein (hereafter referred to as a pathogenic variant). The guide RNA was designed to target exon 11 of *BRCA1* and disrupt its function (sequence 5'-GCAGCATTTGAAAACCCCAA). MCF7 cells were transfected with plasmid containing gRNA, Cas9 protein, and puromycin resistance (Addgene [ID #62988]—pSpCas9(BB)−2A-Puro (PX459) V2.0). Control cells underwent a parallel transfection protocol with a null guide RNA plasmid. CRISPR-Cas9 treated cells were clonally expanded and the predicted CRISPR cleavage site was amplified by PCR (forward 5'- GAAAGGATCCTGGGTGTTTG, reverse 5'- CTTGTTTCCCGACTGTGGTT,) and was Sanger sequenced to identify pathogenic variants. Isogenic lines were cultured in DMEM (1:1; Invitrogen) with 10% (v/v) FBS (Invitrogen) and grown in a humidified atmosphere of 5% CO_2_ at 37 °C.

### RNA interference

Cells were seeded at 5000 cells per well in 96-well tissue culture plates and allowed to adhere overnight. Cells were transfected with 20 µM of Silencer™ Select siRNA oligonucleotides targeting human *SULT1A1* (s13613, Ambion) or a non-targeting siRNA negative control (Negative Control No. 1 siRNA, Ambion). Cells were transfected using Lipofectamine RNAi max (Invitrogen) according to manufacturer’s specifications. After 24 h of transfection media was replaced with normal growth media.

### qPCR

Total RNA was extracted 72 h post transfection using the RNAgem-PLUS kit (ZyGem) to assess the level of gene knockdown. cDNA was synthesized using the Superscript III cDNA Synthesis Kit (Invitrogen) and qPCR was performed using Kapa Probe Fast qPCR Master mix (Kapa Biosystems) on the LightCycler 480 (Roche). The 2^−ΔΔCT^ method was used to quantify mRNA expression levels of target genes, where *HPRT1* was used as an internal reference control. Two well-characterised samples from 1000 Genomes Project with known copy number status were used as copy number controls. Gene-specific primers and fluorescent probes are reported in Supplementary Data 17. Statistical significance was assessed by two-tailed Student’s *t*-tests between target genes and the siRNA control. Expression differences were considered statistically significant if the *p*-value was <0.05.

### Proliferation assay

MCF7 and MCF7–*BRCA1*^+/−^ cells were seeded at 5000 cells per well in 96-well, black walled, clear-bottom tissue culture plates (Greiner). Cells were allowed to adhere overnight before transfection. Media was replaced 24 h post transfection. Forty-eight hours post media change, cell proliferation was assessed using the CyQUANT™ Cell Proliferation Assay Kit (Invitrogen) according to the manufacturer’s instructions. Fluorescence was measured on the Varioskan® Flash plate reader (Thermo Fisher Scientific) using a filter combination for excitation at 480 nm and emission at 520 nm.

### DNA damage assay

Cells were seeded at 50,000 cells per well in 24-well tissue culture plates. Cells were allowed to adhere overnight and were transfected for 24 h before media was replaced. A further 24 h after media replacement cells were treated with 1 µM 4-hydroxyestradiol (4-OHE2, Sigma) or 10 µM Mitomycin C (MMC, Sigma) for 3 h. Cells were gently washed with PBS and media was replaced with fresh complete media for a further 21 h before being assayed for DNA damage.

### Immunocytochemistry

Cells were gently lifted from cell culture plates, cytospun onto slides, and fixed in ice-cold absolute methanol for 5 min. Slides were washed with PBS and blocked for 30 min with 1% bovine serum albumin in PBS-T (Tween-20 0.1% v/v). Slides were dual stained for 1 h with the mouse anti-phospho-H2AX (Ser139) antibody (1:500; ab26350, Abcam) and rabbit anti-53BP1 (1:500; ab36823, Abcam). Slides were incubated with anti-mouse AlexaFluor 488-conjugated (1:400; ab150113, Abcam) and anti-rabbit IgG-AlexaFluor 494-conjugated (1:400; ab150080, Abcam) secondary antibodies, and stained with DAPI for microscopic examination. Images were taken at 40 × magnification on the Axio Imager.Z1 Microscope (Zeiss). Co-localised ϒ-H2AX and 53BP1 foci were counted in >150 cells from a minimum of ten fields from three independent experiments.

### Comet assay

Alkaline comet assays were performed using a comet assay kit (AbCam). Harvested cells were mixed with low melting agarose and transferred onto a glass slide covered in a base layer of agarose. Slides were immersed in lysis buffer for 60 min at 4 °C. Lysis buffer was replaced with alkaline solution (300 mM NaOH, pH 10, 1 mM EDTA) and samples were kept in the dark for 30 min. Slides were transferred to an electrophoresis chamber filled with alkaline solution and electrophoresis was performed for 20 min (1 V/cm). DNA was stained with Vista Green DNA Dye and images were captured by fluorescence microscopy on the Axio Imager.Z1 Microscope. Comets were scored using the CellProfiler software v3.1.8^69^. Tail moments were assessed for >100 cells in three independent experiments.

### Statistical analysis of in vitro data

All in vitro data were expressed as the mean ± standard error. The normality of data was visualised using the Q–Q plot and tested using the Shapiro–Wilk normality test. Statistical significance of differences between control and test groups were determined by an unpaired Student’s *t*-test or two-way analysis of variance (ANOVA). All statistical tests were two sided and *p*-values < 0.05 were considered significant.

### Reporting summary

Further information on research design is available in the Nature Research Reporting Summary linked to this article.

## Supplementary information

Peer Review File

Supplementary Information

Description of Additional Supplementary Files

Supplementary Data 1–17

Reporting Summary

## Data Availability

Genome-wide association summary statistics are available within the article. CIMBA phenotype data used in this study from BCFR-AU, BCFR-NC, BCFR-NY, BCFR-PA, BCFR-UT, BFBOCC, BIDMC, BMBSA, CBCS, CNIO, COH, DEMOKRITOS, DFCI, FCCC, GEORGETOWN, HCSC, HRBCP, HUNBOCS, HVH, ICO, KCONFAB, KUMC, MAYO, MSKCC, MUV, NCI, NNPIO, NORTHSHORE, OSUCCG, PBCS, SMC, SWE-BRCA, UCHICAGO, UCSF, UPENN, UPITT, UTMDACC, VFCTG, and WCP studies are available in the dbGaP database under accession code phs001321.v1.p1. The complete dataset is not publicly available due to restraints imposed by the ethical committees of individual studies. Requests to access the complete dataset, which is subject to General Data Protection Regulation (GDPR) rules, can be made to the Data Access Coordinating Committee (DACC) of CIMBA, following the process described on the CIMBA website (http://cimba.ccge.medschl.cam.ac.uk/projects/data-access-requests/). Submitted applications are reviewed by the CIMBA DACC every 3 months. CIMBA DACC approval is required to access data from studies BCFR-ON/OCGN, BFBOCC-LV, BRICOH, CCGCRN, BRICOH, CONSIT TEAM, DKFZ, EMBRACE, FPGMX, GC-HBOC, GEMO, G-FAST, HEBCS, HEBON, IHCC, ILUH, INHERIT, IOVHBOCS, IPOBCS, KOHBRA, MCGILL, NCCS, NRG_ONCOLOGY, OUH, SEABASS, and UKGRFOCR (see Supplementary Data 12 —for a list of all CIMBA studies). Summary statistics for each GWAS conducted for this study, can be freely downloaded from the NHGRI-EBI GWAS catalogue with the accession codes: GCST90134567; GCST90134568; GCST90134569; and GCST90134570; (https://www.ebi.ac.uk/gwas/). The source data for all figures are presented in the Supplementary Data file.

## Code availability

Code for the retrospective likelihood analysis of variants is available on request.
