# Supplementary material for: Copy number variants as modifiers of breast cancer risk for BRCA1/BRCA2 pathogenic variant carriers
Source: Commun Biol. 2022 Oct 6;5:1061. doi: 10.1038/s42003-022-03978-6 (PMC9537519; doi:10.1038/s42003-022-03978-6)

SUPPLEMENTARY INFORMATION

Supplementary Figure 1: Location of CNVs validated by TaqMan 2

Supplementary Figure 2: Validation of copy number deletions overlapping *SULT1A1* using multiplex ligation-dependent probe amplification 3

Supplementary Figure 3: Representative images of comet assay for MCF7-C1 and MCF7-*BRCA1*$^{+/-}$ cells treated with 4-hydroxyestradiol 4

Supplementary Figure 4: Representative images of comet assay for MCF7-C1 and MCF7-*BRCA1*$^{+/-}$ cells treated with Mitomycin C 5

Supplementary Figure 5: Representative immunocytochemistry images of transfected and MCF7-C1 and MCF7-*BRCA1*$^{+/-}$ cells treated with 4-hydroxyestradiol..6

Supplementary Figure 6: Representative immunocytochemistry images of transfected MCF7-C1 and MCF7-*BRCA1*$^{+/-}$ cells treated with Mitomycin C 8

Supplementary Figure 7: Quality control and filtering of study cohort..10

Supplementary Figure 1: Location of CNVs validated by TaqMan

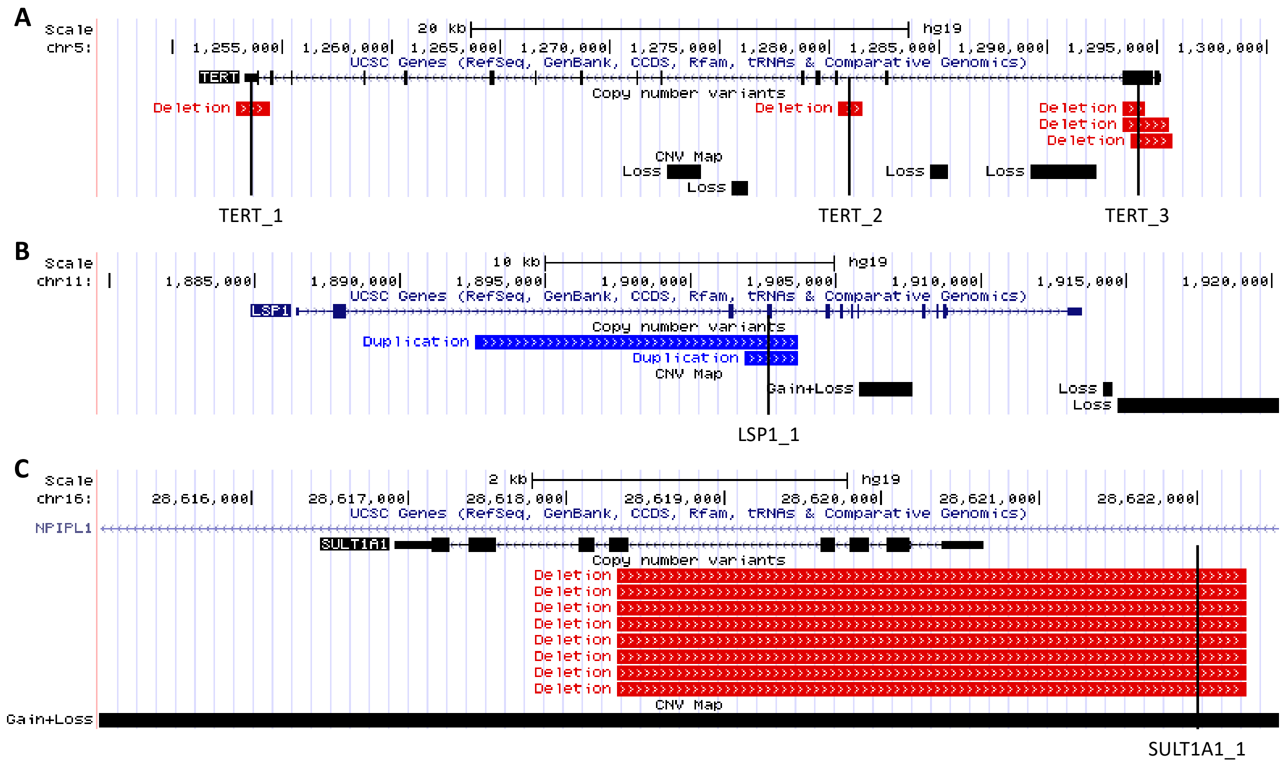

Location of copy number variants available for validation. Genomic view of the **a** *TERT*, **b** *LSP1*, and **c** *SULT1A1* gene loci. View showing UCSC RefSeq gene regions used for association analysis with predicted copy number deletions (red) and duplications (blue), and the Zarrei et al (2015) CNV map.

Supplementary Figure 2: Validation of copy number deletions overlapping *SULT1A1* using multiplex ligation-dependent probe amplification

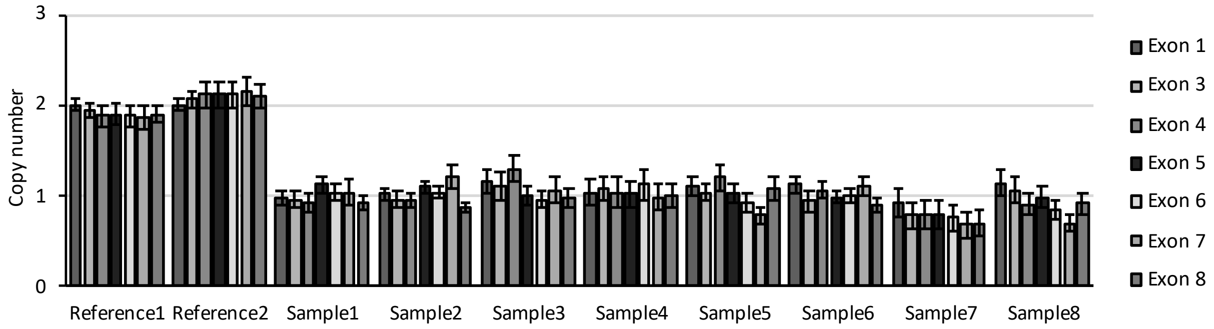

Validation of copy number deletions overlapping *SULT1A1* using multiplex ligation dependent probe amplification. Copy number of the eight exons of the *SULT1A1* reference transcript for eight samples, shown relative to two reference samples with known diploid copy number status.

Supplementary Figure 3: Representative images of comet assay for MCF7-C1 and MCF7-*BRCA1*$^{+/-}$ cells treated with 4-hydroxyestradiol

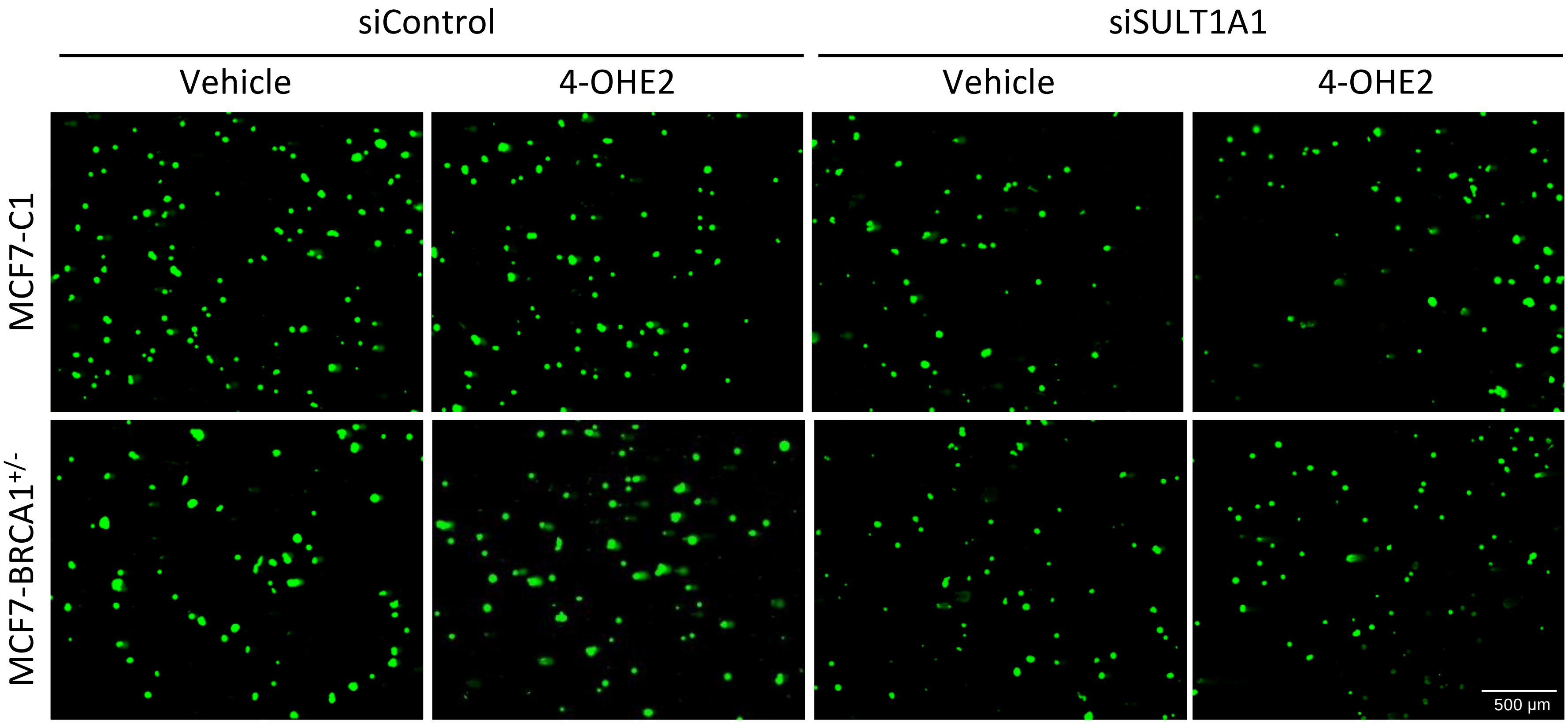

Representative images of comet assay for MCF7-C1 and MCF7-*BRCA1*$^{+/-}$ cells treated with 4-hydroxyestradiol. MCF7-C1 (top) and MCF7-*BRCA1*$^{+/-}$ cells (bottom) were transfected with either siControl (left) or siSULT1A1 (right) for 24 hours and treated, and then treated with either 1 µm 4-hydroxyestradiol (4-OHE2) or the vehicle control for 3 hours. Cells were assayed 21 hours after removal of drug treatment. Images shown were taken at the 20x objective. Scale bar = 500µm.

Supplementary Figure 4: Representative images of comet assay for MCF7-C1 and MCF7-*BRCA1*$^{+/-}$ cells treated with Mitomycin C

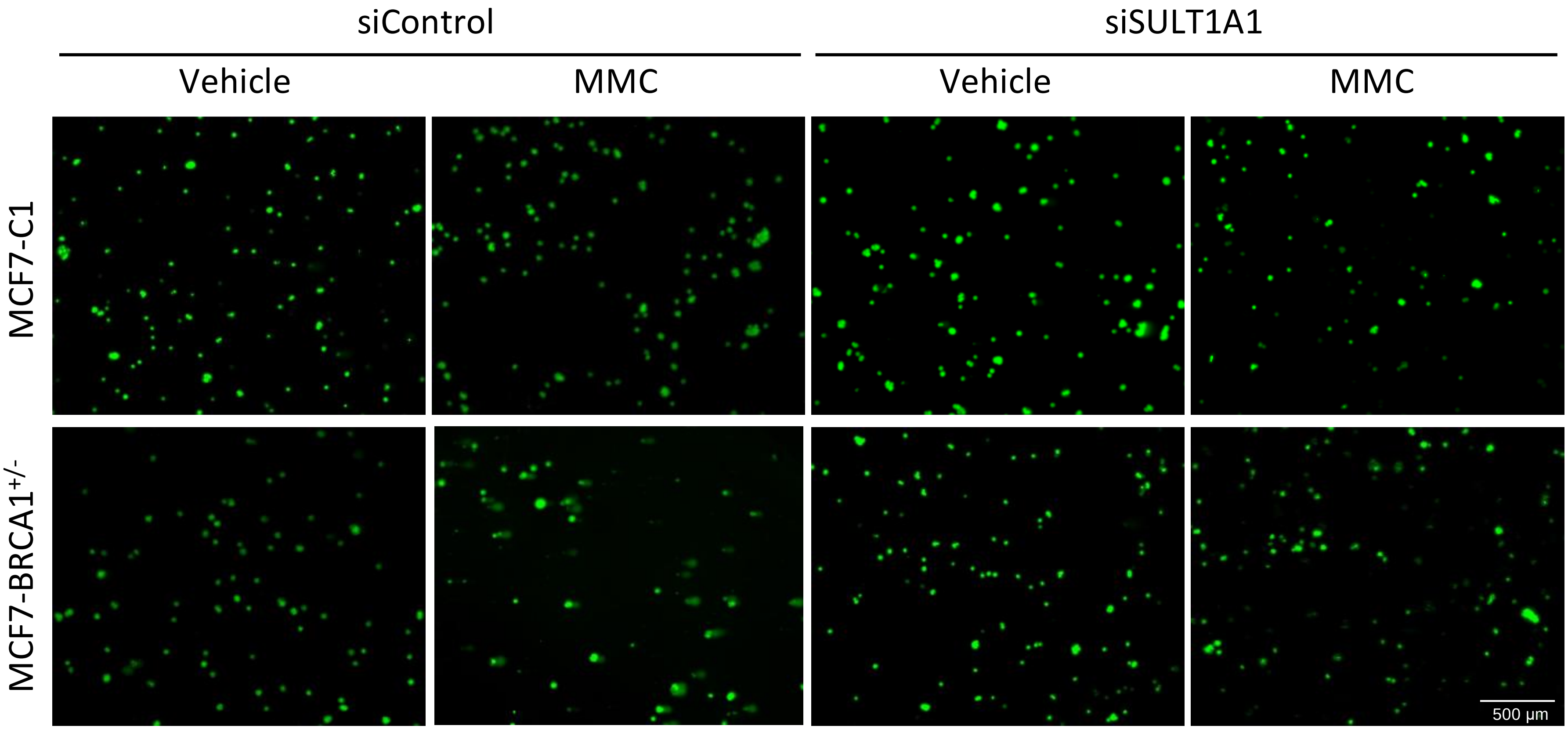

Representative images of comet assay for MCF7-C1 and MCF7-BRCA1$^{+/-}$ cells treated with Mitomycin C. MCF7-C1 (top) and MCF7-BRCA1$^{+/-}$ cells (bottom) were transfected with either siControl (left) or siSULT1A1 (right) for 24 hours and treated, and then treated with either 10 µm Mitomycin C (MMC) or the vehicle control for 3 hours. Cells were assayed 21 hours after removal of drug treatment. Images shown were taken at the 20x objective. Scale bar = 500µm.

Supplementary Figure 5: Representative immunocytochemistry images of transfected and MCF7-C1 and MCF7-*BRCA1*$^{+/-}$ cells treated with 4-hydroxyestradiol

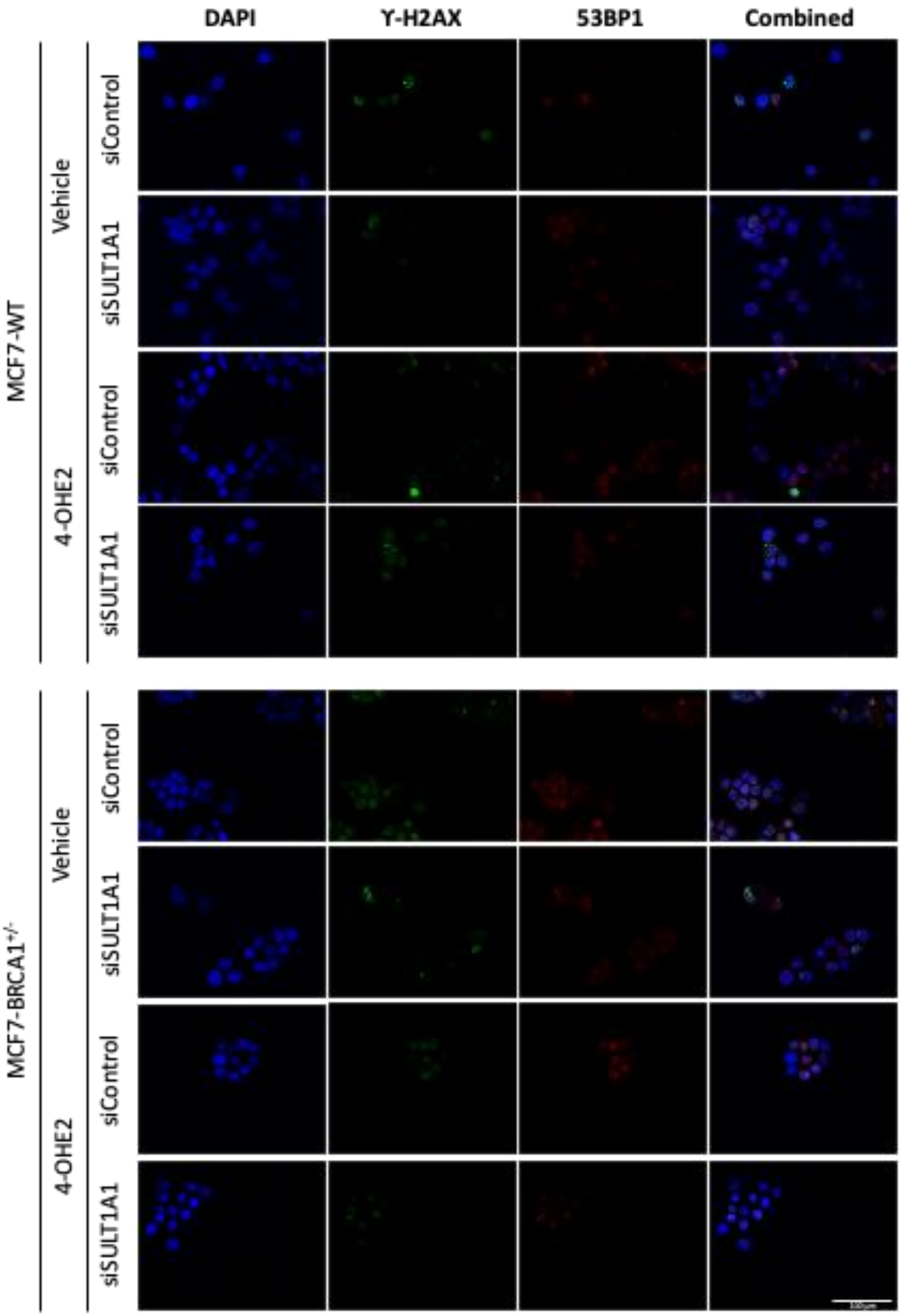

Representative images of immunoassayed MCF7-C1 (top) and MCF7-*BRCA1*$^{+/-}$ cells (bottom). Cells were transfected with either siSULT1A1 or siControl for 24 hours and treated with either 1 μm 4-hydroxyestradiol (4-OHE2) or the vehicle

control for 3 hours. Cells were immunostained with DAPI (blue) gamma-H2AX (green) and 53BP1 (red) 21 hours after removal of drug treatment. Images were taken at the 40x objective. Scale bar = 100μm.

Supplementary Figure 6: Representative immunocytochemistry images of transfected MCF7-C1 and MCF7-*BRCA1*$^{+/-}$ cells treated with Mitomycin C

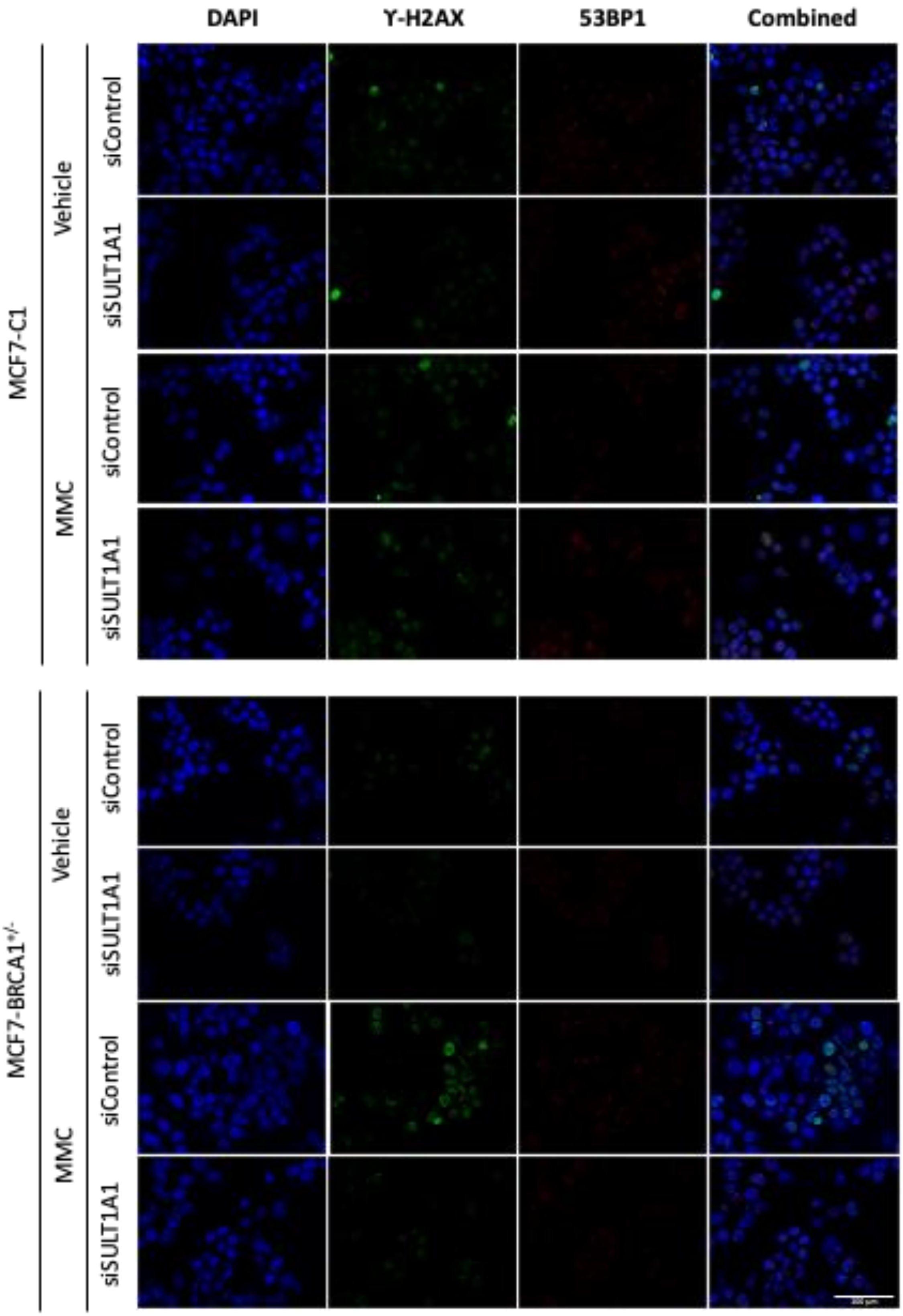

Representative images of immunoassayed MCF7-C1 (top) and MCF7-*BRCA1*$^{+/-}$ cells (bottom). Cells were transfected with either siSULT1A1 or siControl for 24 hours and treated with either 10 μm Mitomycin C (MMC) or the vehicle

control for 3 hours. Cells were immunostained with DAPI (blue) gamma-H2AX (green) and 53BP1 (red) 21 hours after removal of drug treatment. Images were taken at the 40x objective. Scale bar = 100µm.

Supplementary Figure 7: Quality control and filtering of study cohort

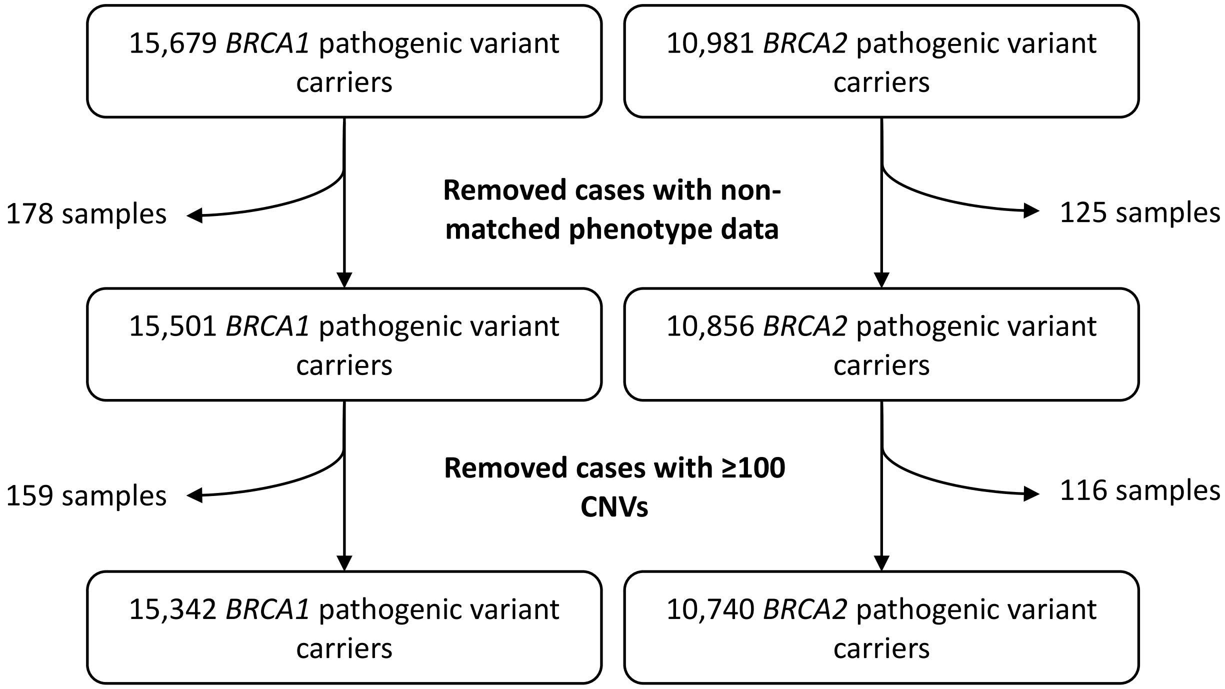

Supplement: Supplementary file 2 — Supplementary Information [file 42003_2022_3978_MOESM2_ESM.pdf]
