# Supplementary material for: Copy number variants as modifiers of breast cancer risk for BRCA1/BRCA2 pathogenic variant carriers
Source: Commun Biol. 2022 Oct 6;5:1061. doi: 10.1038/s42003-022-03978-6 (PMC9537519; doi:10.1038/s42003-022-03978-6)
Supplement: Supplementary file 3 — Description of Additional Supplementary Files [file 42003_2022_3978_MOESM3_ESM.pdf]

Description of Additional Supplementary Files

File name: Supplementary Data 1
Description: Source data for Figure 2 panels b-f

File name: Supplementary Data 2
Description: Source data for Figure 3 panels a-h

File name: Supplementary Data 3
Description: Sensitivity and specificity of calling BRCA1 and BRCA2 copy number variants

File name: Supplementary Data 4
Description: Copy number variants identified by whole-genome sequencing

File name: Supplementary Data 5
Description: Proportion of copy number variant called by PennCNV that were supported by whole-genome sequencing

File name: Supplementary Data 6
Description: Proportion of copy number variants that were not supported by whole-genome sequencing that were supported by the CNV map

File name: Supplementary Data 7
Description: Associations (unadjusted $p<0.01$) with breast cancer risk for deletions overlapping gene regions for BRCA1 pathogenic variant carriers

File name: Supplementary Data 8
Description: Associations (unadjusted $p<0.01$) with breast cancer risk for deletions overlapping gene regions for BRCA2 pathogenic variant carriers

File name: Supplementary Data 9
Description: Associations (unadjusted $p<0.01$) with breast cancer risk for duplications overlapping gene regions for BRCA1 pathogenic variant carriers

File name: Supplementary Data 10
Description: Associations (unadjusted $p<0.01$) with breast cancer risk for duplications overlapping gene regions for BRCA2 pathogenic variant carriers

File name: Supplementary Data 11
Description: Breast cancer hazard ratio estimates for deletions and duplications in BRCA1 or BRCA2 using the score test

File name: Supplementary Data 12
Description: Participating CIMBA studies

File name: Supplementary Data 13
Description: List of BRCA1/2 pathogenic variants in study participants (provided as a separate file)

File name: Supplementary Data 14
Description: Study cohort characteristics for 15,342 BRCA1 and 10,740 BRCA2 pathogenic variant carriers.

File name: Supplementary Data 15
Description: Assay primer and probe sequences, or proprietary design

File name: Supplementary Data 16
Description: Whole-genome sequencing metrics

File name: Supplementary Data 17
Description: RT-qPCR assays
