# Supplementary material for: Copy number variants as modifiers of breast cancer risk for BRCA1/BRCA2 pathogenic variant carriers
Source: Commun Biol. 2022 Oct 6;5:1061. doi: 10.1038/s42003-022-03978-6 (PMC9537519; doi:10.1038/s42003-022-03978-6)
Supplement: Supplementary file 5 — Reporting Summary [file 42003_2022_3978_MOESM5_ESM.pdf]

nature research

Corresponding author(s): Logan C Walker
Last updated by author(s): Aug 16, 2022

Reporting Summary

Nature Research wishes to improve the reproducibility of the work that we publish. This form provides structure for consistency and transparency in reporting. For further information on Nature Research policies, see our Editorial Policies and the Editorial Policy Checklist.

Statistics

For all statistical analyses, confirm that the following items are present in the figure legend, table legend, main text, or Methods section.

n/a	Confirmed	
☐	☒	The exact sample size (n) for each experimental group/condition, given as a discrete number and unit of measurement
☐	☒	A statement on whether measurements were taken from distinct samples or whether the same sample was measured repeatedly
☐	☒	The statistical test(s) used AND whether they are one- or two-sided<br>*Only common tests should be described solely by name; describe more complex techniques in the Methods section.*
☐	☒	A description of all covariates tested
☐	☒	A description of any assumptions or corrections, such as tests of normality and adjustment for multiple comparisons
☐	☒	A full description of the statistical parameters including central tendency (e.g. means) or other basic estimates (e.g. regression coefficient) AND variation (e.g. standard deviation) or associated estimates of uncertainty (e.g. confidence intervals)
☐	☒	For null hypothesis testing, the test statistic (e.g. F, t, r) with confidence intervals, effect sizes, degrees of freedom and P value noted<br>*Give P values as exact values whenever suitable.*
☒	☐	For Bayesian analysis, information on the choice of priors and Markov chain Monte Carlo settings
☒	☐	For hierarchical and complex designs, identification of the appropriate level for tests and full reporting of outcomes
☒	☐	Estimates of effect sizes (e.g. Cohen's d, Pearson's r), indicating how they were calculated

Our web collection on statistics for biologists contains articles on many of the points above.

Software and code

Policy information about availability of computer code

Data collection	No software was used
Data analysis	CNV association analysis - R (V3.3.1) and bespoke software (available on request); MLPA - Coffalyser software (v9.4); Next generation sequencing - Lumpy (V0.2.13) , CNVnator (V0.3.3), Bcl2fastq (V2.16), BWA-mem (V0.7.10-r789), Novosort (V1.03.01) and GATK (V3.3).

For manuscripts utilizing custom algorithms or software that are central to the research but not yet described in published literature, software must be made available to editors and reviewers. We strongly encourage code deposition in a community repository (e.g. GitHub). See the Nature Research guidelines for submitting code & software for further information.

Data

Policy information about availability of data

All manuscripts must include a data availability statement. This statement should provide the following information, where applicable:

- Accession codes, unique identifiers, or web links for publicly available datasets
- A list of figures that have associated raw data
- A description of any restrictions on data availability

Genome-wide association summary statistics are available within the article. CIMBA phenotype data used in this study from BCFR-AU, BCFR-NC, BCFR-NY, BCFR-PA, BCFR-UT, BFBOCC, BIDMC, BMBSA, CBCS, CNIO, COH, DEMOKRITOS, DFCI, FCCC, GEORGETOWN, HCSC, HRBCP, HUNBOCS, HVH, ICO, KCONFAB, KUMC, MAYO, MSKCC, MUV, NCI, NNPIO, NORTHSHORE, OSU CCG, PBCS, SMC, SWE-BRCA, UCHICAGO, UCSF, UPENN, UPITT, UTMDACC, VFCTG, and WCP studies are available in the dbGaP database under accession code phs001321.v1.p1. The complete dataset is not publicly available due to restraints imposed by the ethical committees of individual studies. Requests to access the complete dataset, which is subject to General Data Protection Regulation (GDPR) rules, can be made to the Data Access Coordinating Committee (DACC) of CIMBA, following the process described on the CIMBA website (http://cimba.ccge.medschl.cam.ac.uk/projects/data-access-requests/). Submitted applications are reviewed by the CIMBA DACC every 3 months. CIMBA DACC approval is required to access data from studies BCFR-ON/OCGN,

BFBOCC-LV, BRICOH, CCGCRN, BRICOH, CONSIT TEAM, DKFZ, EMBRACE, FPGMX, GC-HBOC, GEMO, G-FAST, HEBCS, HEBON, IHCC, ILUH, INHERIT, IOVHBOCS, IPOBCS, KOHBRA, MCGILL, NCCS, NRG_ONCOLOGY, OUH, SEABASS, and UKGRFOCR (see Supplementary Table 10 —for a list of all CIMBA studies)."
The source data for all figures is presented in the Supplementary Data file.

Field-specific reporting

Please select the one below that is the best fit for your research. If you are not sure, read the appropriate sections before making your selection.

☒ Life sciences ☐ Behavioural & social sciences ☐ Ecological, evolutionary & environmental sciences

For a reference copy of the document with all sections, see nature.com/documents/nr-reporting-summary-flat.pdf

Life sciences study design

All studies must disclose on these points even when the disclosure is negative.

Sample size	A total of 857,647 CNVs carried by 15,342 BRCA1 and 10,740 BRCA2 pathogenic variant carriers were assessed.
Data exclusions	DNA samples were genotyped using the OncoArray-500k BeadChip (Illumina) and standard sample quality control exclusions were performed as previously described for the SNP genotype analysis (Amos et al., 2017; doi: 10.1158/1055-9965.EPI-16-0106).
Replication	We conducted the largest and most comprehensive genome-wide association study of CNVs and breast cancer risk for BRCA1 and BRCA2 pathogenic variant carriers. Due to the uniqueness of the cohort, association findings cannot be replicated at this point in time.
Randomization	BRCA1 and BRCA2 pathogenic variant carriers were sampled with respect to their disease status, therefore we analysed these data by modelling the retrospective likelihood of observing the CNV conditional on the observed phenotype to account for the non-random ascertainment
Blinding	Study participants were selected based on carrier status and were not blinded to the research team

Reporting for specific materials, systems and methods

We require information from authors about some types of materials, experimental systems and methods used in many studies. Here, indicate whether each material, system or method listed is relevant to your study. If you are not sure if a list item applies to your research, read the appropriate section before selecting a response.

Materials & experimental systems

n/a	Involved in the study	
☒	☐	Antibodies
☐	☒	Eukaryotic cell lines
☒	☐	Palaeontology and archaeology
☒	☐	Animals and other organisms
☐	☒	Human research participants
☒	☐	Clinical data
☒	☐	Dual use research of concern

Methods

n/a	Involved in the study	
☒	☐	ChIP-seq
☒	☐	Flow cytometry
☒	☐	MRI-based neuroimaging

Eukaryotic cell lines

Policy information about cell lines

Cell line source(s)	MCF-7
Authentication	Cells showed immunophenotype (e.g. ER and cytokeratin status) consistent with MCF-7 cell lines.
Mycoplasma contamination	All cell lines have tested negative after regular testing for mycoplasma
Commonly misidentified lines (See ICLAC register)	*Name any commonly misidentified cell lines used in the study and provide a rationale for their use.*

Human research participants

Policy information about studies involving human research participants

Population characteristics	Female BRCA1 and BRCA2 pathogenic variant carriers were from 64 study centres across North America, Europe, and Australia participating in the CIMBA consortium

Recruitment	Eligibility criteria for study participants included: (1) female carriers of BRCA1 or BRCA2 pathogenic variants; and (2) minimum 18 years of age at recruitment. Study participants were recruited by 64 study sites.
Ethics oversight	Every contributing study site had their own ethics approval process. Ethics approval for this study was obtained from the University of Otago Ethics Committee (H14/131 and H17/078)

Note that full information on the approval of the study protocol must also be provided in the manuscript.
